# Activator protein transcription factors coordinate human IL-33 expression from noncanonical promoters in chronic airway disease

**DOI:** 10.1172/jci.insight.174786

**Published:** 2024-03-08

**Authors:** Heather E. Raphael, Ghandi F. Hassan, Omar A. Osorio, Lucy S. Cohen, Morgan D. Payne, Ella Katz-Kiriakos, Ishana Tata, Jamie Hicks, Derek E. Byers, Bo Zhang, Jen Alexander-Brett

**Affiliations:** 1 Department of Medicine, Division of Pulmonary and Critical Care Medicine,; 2 Department of Developmental Biology, and; 3 Department of Pathology and Immunology, Washington University School of Medicine, St. Louis, Missouri, USA.

**Keywords:** Immunology, Pulmonology, COPD, Cytokines, Transcription

## Abstract

IL-33 is a cytokine central to type 2 immune pathology in chronic airway disease. This cytokine is abundantly expressed in the respiratory epithelium and increased in disease, but how expression is regulated is undefined. Here we show that increased *IL33* expression occurs from multiple noncanonical promoters in human chronic obstructive pulmonary disease (COPD), and it facilitates production of alternatively spliced isoforms in airway cells. We found that phorbol 12-myristate 13-acetate (PMA) can activate *IL33* promoters through protein kinase C in primary airway cells and lines. Transcription factor (TF) binding arrays combined with RNA interference identified activator protein (AP) TFs as regulators of baseline and induced *IL33* promoter activity. ATAC-Seq and ChIP-PCR identified chromatin accessibility and differential TF binding as additional control points for transcription from noncanonical promoters. In support of a role for these TFs in COPD pathogenesis, we found that AP-2 (*TFAP2A*, *TFAP2C*) and AP-1 (*FOS* and *JUN*) family members are upregulated in human COPD specimens. This study implicates integrative and pioneer TFs in regulating *IL33* promoters and alternative splicing in human airway basal cells. Our work reveals a potentially novel approach for targeting IL-33 in development of therapeutics for COPD.

## Abstract 2

Baseline and activated IL-33 expression can be regulated in the respiratory epithelium and these pathways are dysregulated in chronic airway disease.

## Graphical abstract

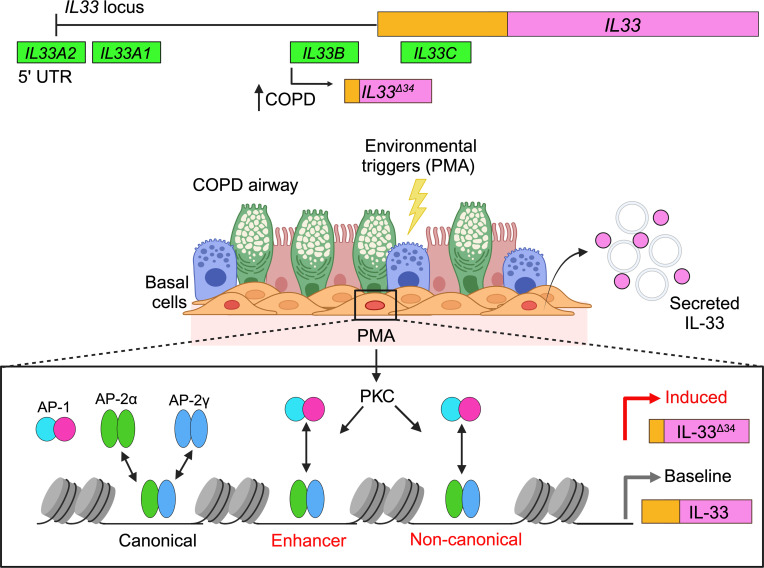

## Introduction

Cytokines play a central role in amplification and propagation of inflammation in chronic obstructive pulmonary disease (COPD) and asthma (1), making them primary targets in the development of biologic therapeutics (2). A role for IL-33 in human airway disease pathogenesis was established by a series of large-scale genome-wide association studies (GWAS) reporting a link between asthma and single-nucleotide polymorphisms (SNPs) flanking the *IL33* and *IL1RL1/ST2* (IL-33 signaling receptor) loci (3–5). IL-33 is a stimulus for both innate and adaptive type 2 immune programs in allergic lung inflammation (reviewed in refs. 6, 7) and an especially potent stimulus for IL-13 production by type 2 innate lymphoid cells (ILC2) (8–10). IL-33 also promotes mast cell survival and cytokine production (11), as well as eosinophil development (12), that contribute to chronic airway disease.

Previously, it was shown that mouse *Il33* can be expressed from alternate promoters on both the tissue and cellular level in mice (13). One of the mouse promoters (denoted as *Il33a*) was constitutively active in fibroblasts and endothelial cells, while no baseline *Il33* expression was observed in either primary myeloid cells or cell lines. In this study, TLR agonists were shown to induce expression of a distinct transcript driven by an alternative promoter (*Il33b*) in multiple cell types, but no spliced isoforms were identified. In the human respiratory system, *IL33* expression has been found to be induced by diverse environmental stimuli including viruses (14, 15), cigarette smoke (16–19), allergens (20, 21), and other inflammatory mediators (reviewed in ref. 22). Multiple human *IL33* promoters are annotated in the genome (hg38); 3 are located ~25 kb and ~5 kb upstream of the first coding exon 2, and 1 is located in the intron between exons 2 and 3, suggesting that there is potential for multiple points of regulation within the human *IL33* gene locus as well.

The majority of baseline *IL33* transcript expression in the lung derives from respiratory epithelial progenitor cells — i.e., basal cells in human and type 2 pneumocytes in mice (15). However, the transcription factors (TFs) regulating baseline and inducible epithelial *IL33* expression under homeostatic and disease conditions are undefined. Truncated spliced *IL33* transcripts have also been cloned from human cell lines (23), asthma endobronchial biopsy samples (24), and primary COPD airway basal cells (25), but the mechanism by which these alternatively spliced forms are produced is also unknown. Truncated isoforms variably lack exons within the N-terminal chromatin interacting domain, some of which are not retained in the nucleus and can be tonically secreted from airway cells (24, 25). We have shown that a spliced *IL33* transcript lacking exons 3 and 4 (*IL33*^Δ34^) is enriched in COPD lung tissues and cultured airway basal cells relative to non-COPD specimens (25). This work prompted us to question what factors could drive expression of pathogenic *IL33* isoforms in COPD.

To address the basis for expression of alternate *IL33* isoforms in airway epithelium, we analyzed transcriptional control of the 4 distinct promoters within the human *IL33* locus. Expression screening revealed that phorbol 12-myristate 13-acetate (PMA) activates multiple *IL33* promoters through protein kinase C (PKC). Further examination of this pathway showed that known downstream targets of PKC, including activator protein-1 (AP-1) and AP-2 TFs regulate baseline and PMA-induced noncanonical *IL33* promoter activity. Based on ATAC-Seq and ChIP-PCR, we found different patterns of accessibility at these promoters and nearby enhancers that tracked with variable *IL33* expression in airway cell lines. These findings are supported by increased expression of AP-2 (*TFAP2A*, *TFAP2C*) and AP-1 (*FOS* and *JUN*) TFs in lung tissue and by nuclear colocalization of IL-33 and phosphorylated AP-2γ protein in COPD airways.

## Results

### IL-33 is expressed from multiple promoters in chronic airway disease.

**Figure 1:**
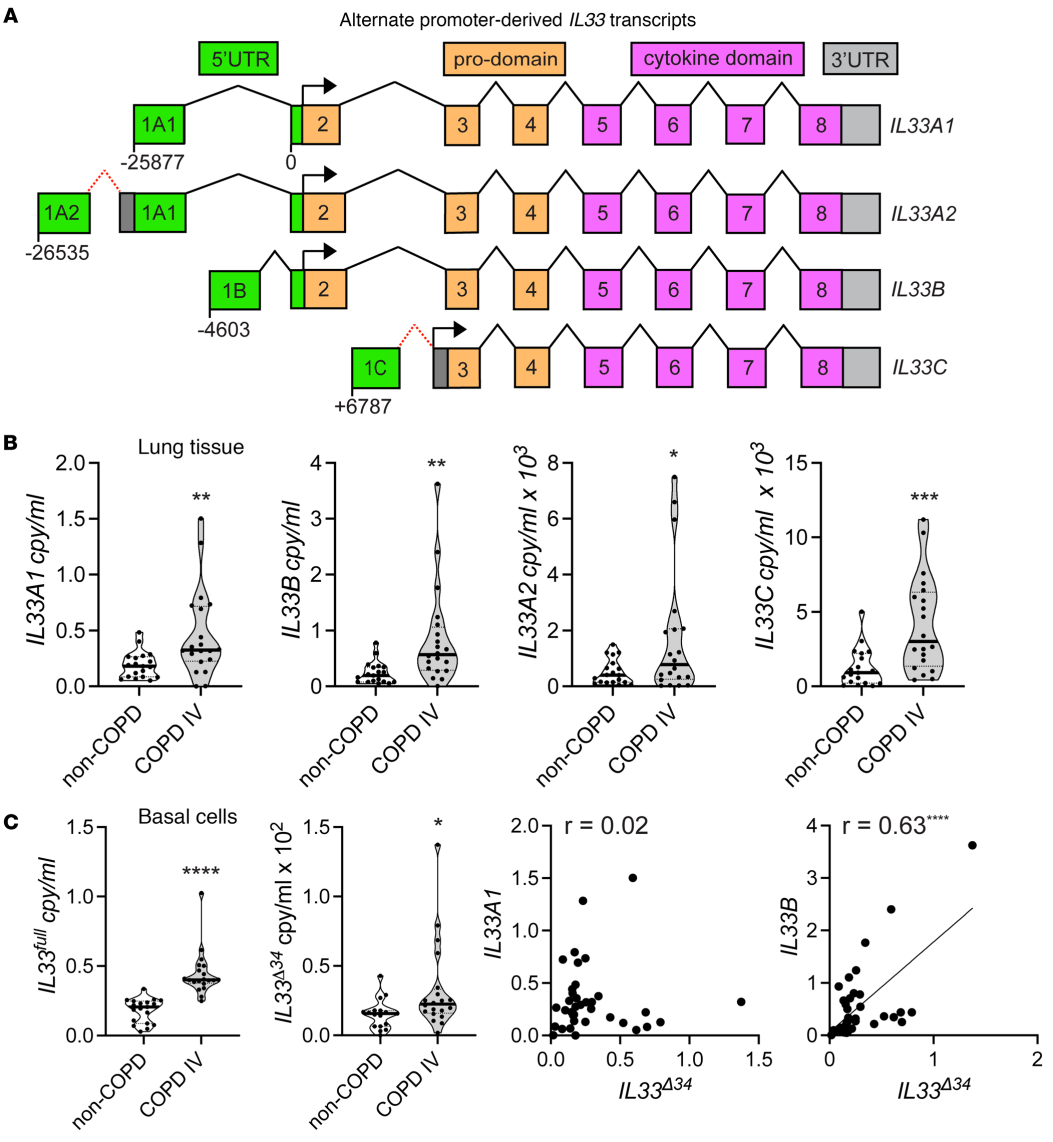
IL-33 expression from multiple promoters in COPD and non-COPD lung tissue. (**A**) Human *IL33* exon structure detailed for promoter-derived transcripts containing unique upstream 5′-noncoding exons 1A (*IL33A*), 1A2 (*IL33A2*), and 1B (*IL33B*) upstream of first coding exon 2 and 1C (*IL33C*) in the intron between exons 2 and 3. (**B**) Transcript mRNA levels measured in lung tissue using isoform-specific qPCR assays quantified from *n* = 18 non-COPD and *n* = 20 COPD specimens; each data point represents an average measurement from 4 different lung regions. (**C**) Transcript mRNA levels measured in cultured airway basal cells quantified from *n* = 12 non-COPD and *n* = 25 COPD specimens. Quantity is displayed as copy/mL and normalized to *GAPDH*. Statistical analysis included *t* test (**B** and **C**), Pearson’s correlation (**C**). *P < 0.05, **P < 0.01, ***P < 0.001. Data is representative of *n* = 2 technical replicates.

We have previously shown that total *IL33* and the truncated isoform *IL33*^Δ34^ are increased in COPD lung tissue specimens and airway basal cells (15, 25). Within the human *IL33* gene, multiple accessioned transcripts are annotated that derive from distinct promoters (Figure 1A). Two close transcription initiation sites are approximately 25 kb upstream of the first coding exon 2, each containing a noncoding exon 1 and resulting transcripts that we denote as *IL33A2* (NM_001314047.2) or *IL33A1* (NM_033439.4). Another transcript is initiated roughly 5 kb upstream of exon 2 (*IL33B*, NM_001314045.2). A fourth transcript begins in the intron between exons 2 and 3 (*IL33C*, NM_001353802.2). These *IL33* isoforms are distinguished by their unique 5′ untranslated (5′UTR) first exon. The *IL33A1* transcript was the first to be accessioned and is generally regarded as the canonical or dominant promoter driving *IL33* expression, largely based on studies in the mouse system (13). However, in some human cell lines, *IL33B* has been shown to be the dominant transcript (26). We were able to clone transcripts designated *IL33A1*, *IL33A2*, and *IL33B* from human COPD airway basal cells and verify the corresponding unique exons 1A1, 1A2, and 1B as depicted in Figure 1A. When cloning with 5′ primers corresponding to the first exon of *IL33A2*, we were only able to isolate a hybrid transcript that was spliced to incorporate both the 5′UTR exons 1A2 and 1A1, including an additional segment between them (Figure 1A and Supplemental Figure 1; supplemental material available online with this article; https://doi.org/10.1172/jci.insight.174786DS1). We were unable to clone a transcript containing only exon 1A2 corresponding to NM_001314047.2. We were also unsuccessful in cloning the *IL33C* isoform from airway basal cells; it has a unique translation start site and protein N-terminus that lacks exon 2 and part of exon 3 (27).

We generated isoform-specific quantitative PCR (qPCR) assays to determine the relative contribution of noncanonical transcripts to total *IL33* expression in COPD tissues and airway basal cells. This analysis includes lung specimens from subjects without COPD and those with severe COPD who underwent lung transplantation (Supplemental Table 1). We validated isoform-specific assays using our cloned transcripts (Supplemental Figure 2) and found that the *IL33A1* qPCR assay detects both *IL33A1* and the *IL33A2* hybrid transcripts based on plasmid standards. qPCR performed on non-COPD and COPD airway tissue demonstrates that all 4 *IL33* transcripts are significantly increased in COPD tissue specimens with a wide range of expression levels observed (Figure 1B). While both *IL33A1* and *IL33B* expression were similar in lung tissue, specific *IL33A2* and *IL33C* expression was 2–3 orders of magnitude lower, indicating that these promoter-driven isoforms make a smaller contribution to total lung *IL33* mRNA. We also quantified full-length *IL33* (*IL33^full^*) and *IL33*^Δ34^ levels for this cohort using previously designed assays (25), which again demonstrated a significant increase in COPD relative to non-COPD controls. We then performed correlation analyses for the more abundant *IL33A1* and *IL33B* transcripts, each with *IL33*^Δ34^ expression in lung tissue; this demonstrated significant association only for *IL33B*. These data show that multiple *IL33* promoters are active in lung tissue with a range of expression levels that are overall higher in COPD specimens. Furthermore, the canonical *IL33A* transcript and noncanonical *IL33B* transcript appear to contribute similarly to total *IL33* expression.

### A noncanonical IL33 transcript contributes to alternative splicing.

**Figure 2:**
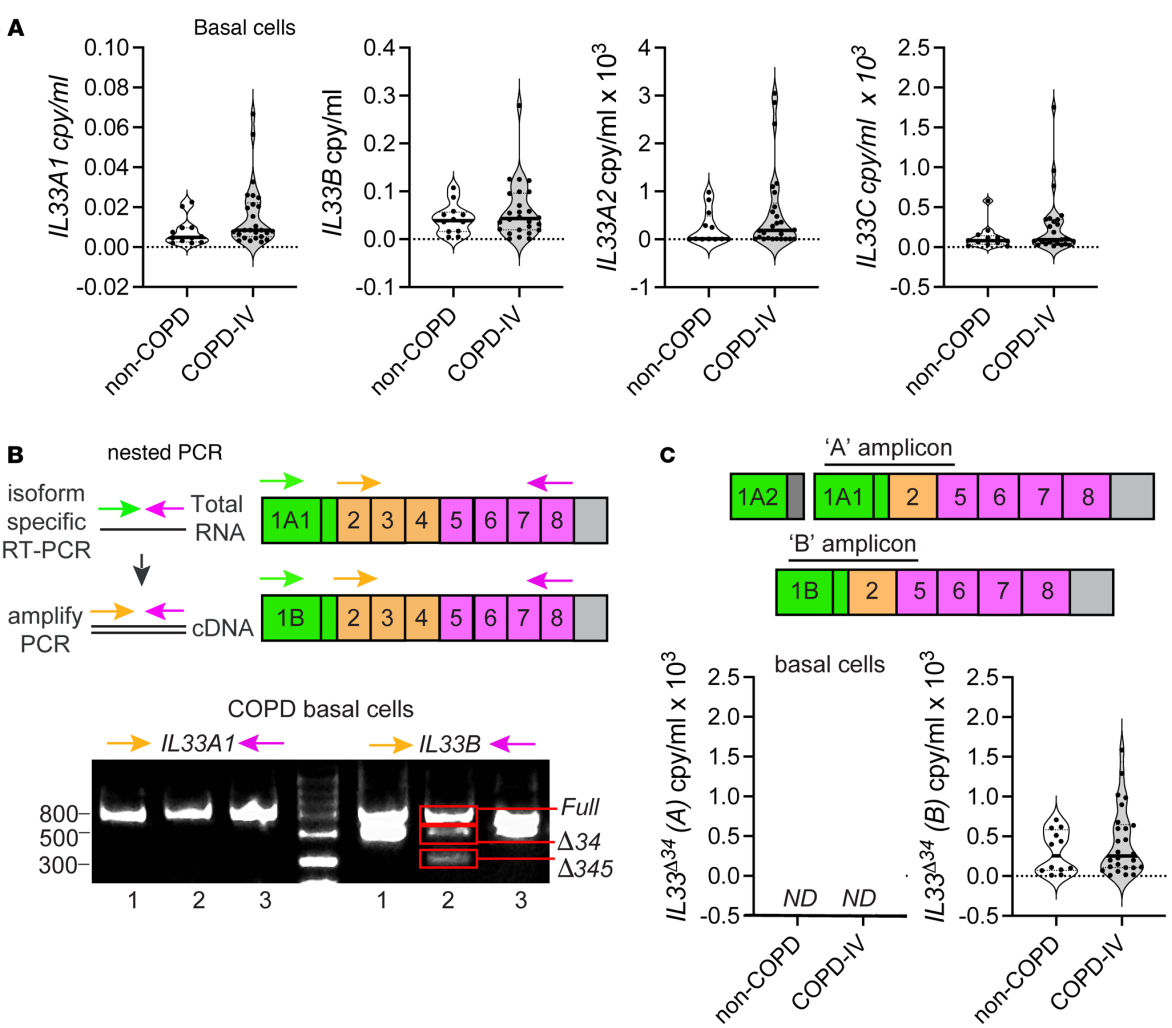
*IL33* spliced isoform is derived from a noncanonical promoter. (**A**) Transcript mRNA levels measured in cultured airway basal cells using promoter-specific qPCR assays as in Figure 1, quantified from *n* = 12 non-COPD and *n* = 25 COPD specimens. (**B**) Nested PCR based on isoform-specific cDNA generation followed by amplification using primers encompassing the *IL33* coding region. Primer sets are color coded according to location, and assay steps are as depicted in the schematic. Agarose gel analysis for a subset of COPD specimens demonstrating truncated bands observed only for PCR amplification on *IL33B*. (**C**) qPCR using primer-probe sets designed to detect the truncated *IL33^Δ34^* transcript using random primer-generated cDNA template from **A**, quantified as copy/mL using plasmid standard and normalized to *GAPDH*. Statistical analysis included *t* test (**A** and **C**). Data in **A** are representative of *n* = 2 technical replicates; experiments in **B** and **C** were repeated in triplicate.

Given the observed correlation between *IL33B* and *IL33*^Δ34^ expression in lung tissue, we hypothesized that truncated spliced isoforms may be preferentially derived from the *IL33B* transcript. Precedent for this concept includes a previous report on *bcl-X*, which exhibits distinct splicing patterns based on interactions between alternate promoter–derived 5′UTR sequences with a common 3′UTR sequence (28). We similarly analyzed a cohort of COPD and non-COPD cultured airway basal cells by qPCR using the same assays as in Figure 1. We found that *IL33A1* and *IL33B* transcripts were both abundantly expressed, with the noncanonical *IL33B* transcript expressed at slightly higher levels relative to the canonical *IL33A1*, and both *IL33A2* and *IL33C* were again 2–3 orders of magnitude lower (Figure 2A). None of the transcripts were significantly increased in COPD basal cells compared with non-COPD in culture, and this may reflect altered promoter activity influenced by culture conditions or passaging of cells.

To test whether IL-33^Δ34^ was differentially generated from transcripts containing either exon 1A1 or 1B, we generated complimentary DNA (cDNA) from COPD basal cells using transcript-specific primer sets including unique 5′ exons 1A1 or 1B and the same 3′ primer corresponding to the protein C-terminus in exon 8 (Figure 2B). We then amplified each reaction with cloning primers specific to the IL-33 coding region (exon 2–8) and analyzed by agarose gel, which demonstrated a single PCR product based on *IL33A1* for the specimens tested but multiple truncated bands for *IL33B* (Figure 2B). We isolated and sequenced these PCR products and found that *IL33A* cDNA yielded only *IL33^full^*, while *IL33B* yielded *IL33^full^*, *IL33*^Δ34^, and *IL33*^Δ345^ spliced products. We then performed qPCR analysis on our cohort of COPD and non-COPD airway basal cells shown in Figure 2A using qPCR assays designed to detect the truncated *IL33*^Δ34^ isoform in the context of *IL33A1* or *IL33B* transcripts (Figure 2C). Specificity was validated for these assays, as shown in Supplemental Figure 2. Similar quantities of *IL33A* and *IL33B* transcripts were amplified in the random-primer generated cDNA libraries per Figure 2A, but the *IL33*^Δ34^ specific assays only amplified on the B amplicon, no signal was detected from the A amplicon. We have previously shown the *IL-33*^Δ34^ isoform can be tonically secreted from airway basal cells (25), and these results provide insight into regulation of truncated spliced isoforms; among these, the *IL-33*^Δ34^ isoform appears to be preferentially generated through noncanonical *IL33B* promoter usage.

### Phorbol esters induce noncanonical IL33 promoters through PKC.

**Figure 3:**
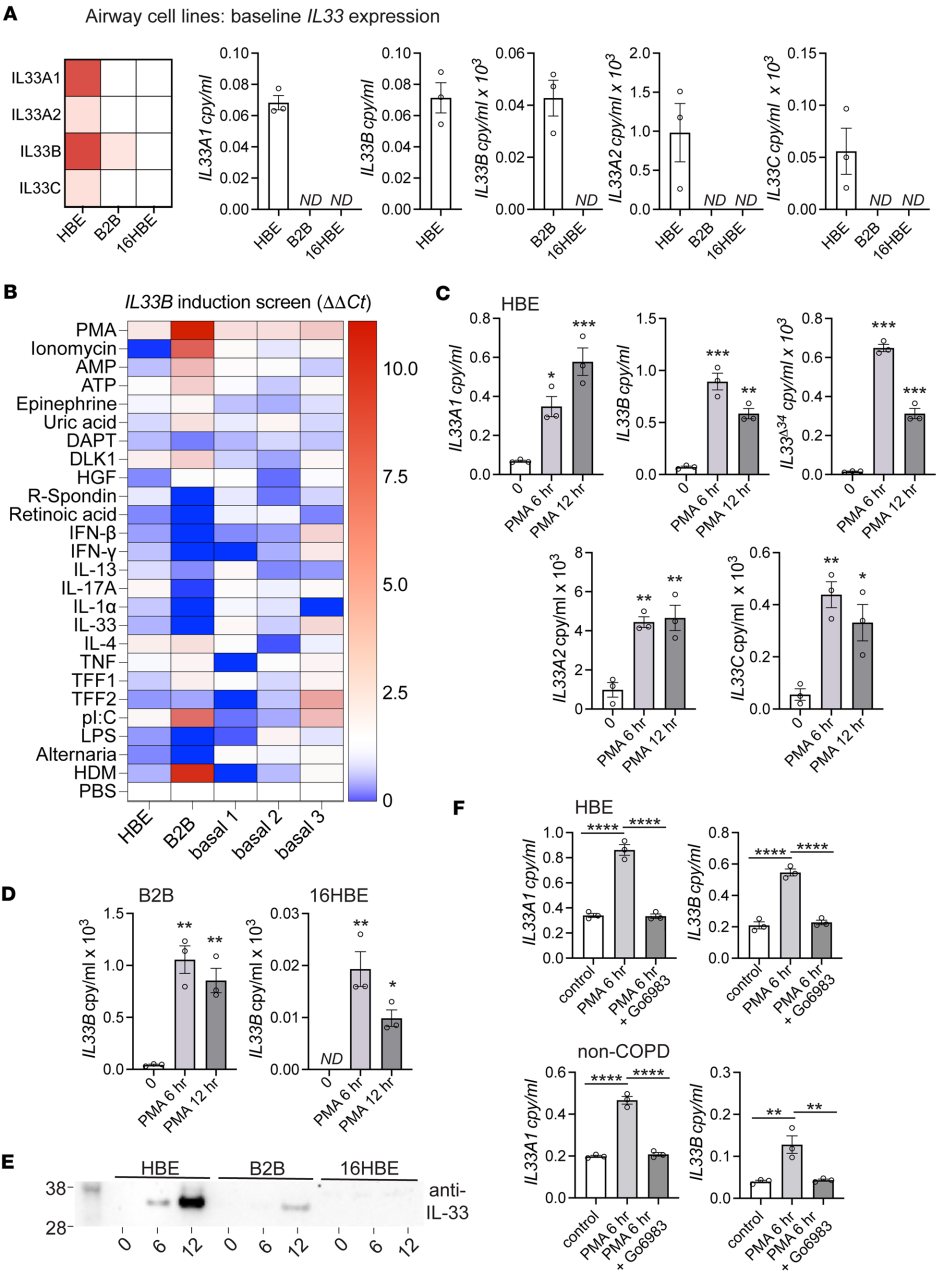
PMA induces *IL33* expression in airway basal cells and lines. (**A**) Baseline expression of *IL33* isoforms as defined in Figure 1; summary heatmap of relative expression is shown to the left. White indicates no detection. (**B**) *IL33B* expression screen (represented as fold change ΔΔCt normalized to *GAPDH*) in HBE and B2B cell lines and 3 non-COPD airway basal cell specimens, demonstrating consistent induction across cell types with PMA (20 ng/mL). (**C**) Time-course PMA treatment in HBE cells demonstrating induction of all promoter-driven isoforms and *IL33^Δ34^*. (**D**) Time course as in **C** performed for B2B and 16HBE cells. (**E**) Protein induction measured by anti-IL33 Western blot (C-terminal domain, R&D systems; Supplemental Table 2) performed on HBE, B2B, and 16HBE cell lysates at 6 and 12 hours after PMA. (**F**) Expression analysis of *IL33A1* and *IL33B* for PMA induction without and with PKC inhibitor Go6983 (500 nM), shown for both HBE cells and non-COPD airway basal cells. Statistical analysis included 1-way ANOVA (**C**, **D**, and **F**),. *P < 0.05, **P < 0.01, ***P < 0.001, ****P < 0.0001. **A** and **C**–**F** were repeated in triplicate. **B** is representative of duplicate technical replicates.

In order to test factors that influence expression from noncanonical IL-33 promoters, we first characterized 3 cell lines that approximate airway basal cells in vitro; HBE-1 (HBE), Beas2B (B2B) and 16HBE14o- (16HBE) (29–31). We observed that HBE expressed high levels of *IL33A* and *IL33B* transcript at baseline, where B2B expressed only *IL33B* at 1,000-fold lower levels, and 16HBE exhibited no detectable *IL33* expression; these results are quantified and summarized with a heatmap in Figure 3A. To identify stimuli capable of inducing *IL33* expression from the 4 promoters, we curated the literature to assemble a panel of reported stimulants of type 2 inflammation or airway basal cell activation, proliferation, or differentiation (Supplemental Table 3). We performed a 12-hour expression screen for HBE and B2B cell lines along with 3 non-COPD low-passage primary airway basal cell specimens. The cDNA library was generated using a cells-to-cDNA kit, and qPCR for promoter-driven isoforms was performed. Neither baseline or induced *IL33A1*, *IL33A2*, or *IL33C* were detectable due to low cDNA yield (not shown); however, *IL33B* expression was sufficient to be analyzed for all 5 specimens. A heatmap representing fold-change *IL33B* expression relative to PBS for each cell type is shown in Figure 3B, which demonstrates that most conditions exhibited variability across specimens. Importantly, comparison of HBE and B2B lines illustrates that the fold-change *IL33B* expression induction was related to baseline expression levels, with high baseline–expressing cells (HBE; primary basal cells) demonstrating lower-magnitude induction relative to low baseline cells (B2B). The only stimulant in this screen that consistently induced *IL33B* in all cell lines and the 3 primary cell specimens was PMA. Follow-up time course analysis performed in HBE, B2B, and 16HBE cell lines at 6 and 12 hours demonstrated reproducible induction after PMA treatment (Figure 3, C and D). In HBE, all 4 *IL33* transcripts were induced by PMA at 6 and 12 hours, though with somewhat different kinetics. We observed *IL33B* expression increased 4-fold at 6 hours and began to decay by 12 hours, while *IL33A1* was increased by 2-fold at 6 hours and increased further at 12 hours after PMA treatment. The kinetics of *IL33^Δ34^* expression mirrored *IL33B*, consistent with our observations that this spliced isoform appears to be preferentially derived from the *IL33B* transcript. The *IL33A2* and *IL33C* transcripts were also induced by PMA in HBE but with expression levels 2–3 orders of magnitude lower and with kinetics similar to *IL33A* and *IL33B*, respectively. In the B2B line, expression of *IL33B* was at a low level at baseline but increased 50-fold following PMA treatment. In 16HBE cells, *IL33B* only reached detectable levels with PMA treatment. To test promoter responses in other cell types, we also treated Jurkat cells with PMA (Supplemental Figure 3). We observed *IL33A* expression detectable at very low levels in Jurkat cells and found that it was not PMA responsive; however, induction of *IL33B* and *IL33A2* was observed and appeared to be PMA specific, as a similar response was not induced with LPS treatment.

On the protein level, the Western blot demonstrated induction of IL-33 protein at 6 and 12 hours after PMA treatment in both HBE and B2B cells (Figure 3E). No detectable IL-33 protein induction was observed for 16HBE. Protein expression levels were higher in HBE versus B2B, reflecting *IL33* expression differences observed by qPCR. Of note, the magnitude of protein induction with PMA at 12 hours appears greater than 2-fold in HBE, suggesting that PMA may exert additional posttranscriptional effects on cellular IL-33 protein levels in airway epithelial cells.

To investigate the mechanism of PMA-induced IL-33 expression, we examined the potential role of PKC, given that phorbol esters can activate both PKC and cAMP pathways (32). We treated HBE, B2B, and primary airway basal cells with the PKC inhibitor Go6983 during PMA stimulation and found that this kinase inhibitor blocked PMA induction of *IL33* expression in both airway cell lines and primary cells, with a return to baseline expression levels (Figure 3F and Supplemental Figure 3B).

Together, these results demonstrate that expression activity from all 4 *IL33* promoters can be induced by PMA through a PKC-dependent pathway. The magnitude of induction appears to be, in part, related to baseline expression level, as seen for the fold-change induction in HBE (high baseline) compared with B2B (low baseline). However, there do also appear to be cell-specific differences in induction behavior for low-baseline cells — for example, in 16HBE, where PMA induction was observed but much more limited compared with B2B, with no protein induction observed. Such factors may also be relevant to the wide variability in expression response observed for the 3 primary basal cell specimens. Last, IL-33 protein levels are robustly increased by PMA in HBE cells, which may be due to combined transcriptional and translational effects.

### AP TFs coordinate noncanonical IL-33 expression.

**Figure 4:**
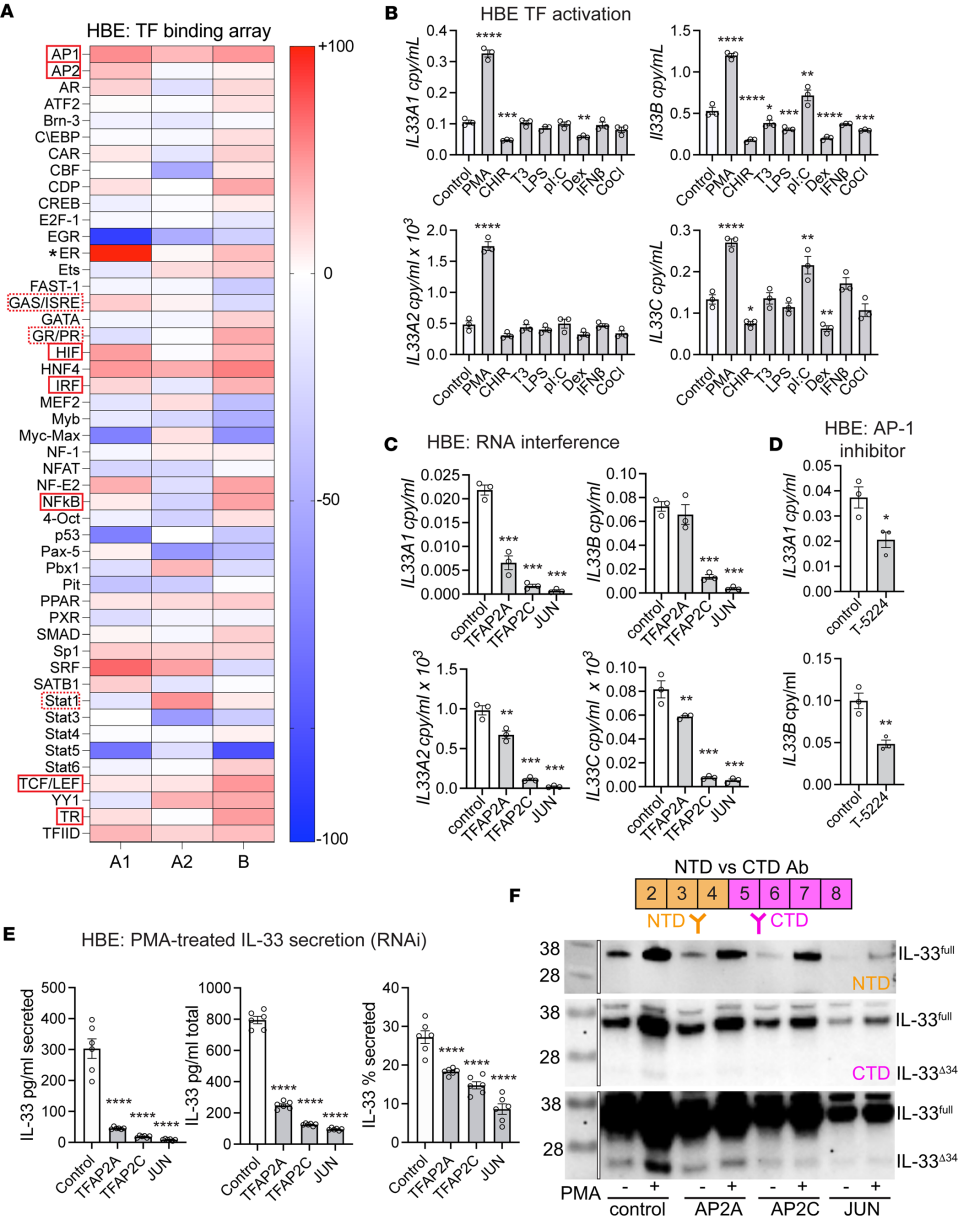
Transcription factor array and follow-up analyses in HBE cells. (**A**) Transcription factor (TF) binding array results for *IL33* A1, A2, and B promoter sequences (600 bp upstream of transcription start site) for HBE cells treated with PMA for 6 hours. Percent inhibition is calculated relative to no-input control luciferase signal, expressed as percent reduction in relative luminescence units. Red indicates decreased luciferase signal, and bule represents increased signal compared with no-input control. TFIID is a positive control for RNA-polymerase transcription initiation complex indicating promoter function. Solid red boxes indicate shared TF hits; dashed red boxes indicate apparent unique hits. (**B**) Follow-up *IL33* expression analysis with 6-hour treatment for candidate transcription factor activators identified from array; see Supplemental Figure 4 for cross-referenced TF expression. Activator concentrations are reported in Supplemental Table 4. (**C**) Expression for *IL33A1*, *IL33B*, *IL33A2*, and *IL33C* with lentiviral mediated RNA interference for *TFAP2A*, *TFAP2C*, and *JUN* in HBE cells. See Supplemental Figure 5 for knockdown validation. (**D**) HBE *IL33A1* and *IL33B* expression in cells treated with AP-1 inhibitor T-5224; concentration in Supplemental Table 4. (**E**) Endogenous IL-33 protein secretion and total protein levels measured under RNA interference conditions with 12-hour PMA treatment, quantified by ELISA. (**F**) Anti–IL-33 Western blot performed under conditions of RNA interference and 12-hour PMA treatment, using both N-terminal domain (NTD) and C-terminal domain (CTD) targeting antibodies. Bands consistent with full-length (IL-33^full^) and IL-33^Δ34^ are labeled; MW fragment ~24 kDa is only reactive with CTD antibody. Markers were run on the same gel for each Western blot; they were cropped for the figure and are indicated by a white line with black border. Statistical analysis included 1-way ANOVA (**B**, **C**, and **E**) and *t* test (**D**). *P < 0.05, **P < 0.01, ***P < 0.001, ****P < 0.0001. **A** represents a single experiment; **B**–**F** are representative of triplicate repeats.

To investigate the TFs responsible for PMA-induced IL-33 expression, we performed a competition-based TF binding array (Signosis). We amplified the A1, A2, and B core promoter regions (600 bp upstream of exons 1A1, 1A2, and 1B) from HBE cells and verified by Sanger sequencing that they matched the hg38 reference genome. We then used these PCR products as competitive DNA in the TF array to assess binding to a panel of 48 TFs in HBE cells following 6 hours of PMA treatment (Figure 4A); readout is the percent inhibition of binding to consensus DNA. Results demonstrate Transcription Factor IID (TFIID, part of RNA polymerase II pre-initiation complex) positive control binding to each A1, A2, and B promoters. The array also yielded several TFs that exhibited binding to 1 or more *IL33* promoter sequences, with some apparently shared and some unique to a given promoter. Among the TFs included in the array, AP-1 and AP-2 TFs were of particular interest because they are known to be activated downstream of PMA and PKC (32, 33).

To identify candidates for follow-up analysis, we cross-referenced TF array hits to airway basal cell expression in reference to other human lung structural cells, using available single-cell RNA-Seq data from the LungMAP consortium (https://app.lungmap.net). Expression analysis revealed that AP-1 and AP-2 family members were among the TFs with the highest expression in airway basal cells of those included in the array (Supplemental Figure 4). To comprehensively address both shared- and differential-binding TFs, we focused follow-up analysis on TFs that exhibited more than 30% inhibition in the assay with binding to 2 or more promoters (AP-1, AP-2, HIF, IRF, NF-κB, TCF/LEF, and TR; red boxes in Figure 4A) or apparent preferential binding to an individual promoter (GAS/ISRE, GR/PR, STAT1; dashed red boxes in Figure 4A). Of note, potent and highly specific inhibition of estrogen receptor (ER; ESR1) binding was observed only for the A1 promoter (Figure 4A). This may reflect the female source of the HBE cell line, while the B2B and 16HBE lines are derived from male donors. This observation was not pursued in the current follow-up assays because expression of ESR1 in airway basal cells could not be confirmed based on LungMAP single-cell data (Supplemental Figure 4) or qPCR in HBE cells (not shown).

When we tested a panel of TF activators in high-baseline HBE and primary basal cells, we found that, among the agents tested — dexamethasone (GR), IFN-β (STAT1, IRF, GAS/ISRE), LPS, poly I:C (NF-κB), T3 (TR), or CHIR99021 (TCF/LEF) — none increased *IL33A* or *IL33B* expression to the same degree as PMA, though a more modest *IL33B* induction was observed for polyI:C in HBE (Figure 4B). Some activators resulted in a significant decrease in expression for multiple promoter-derived transcripts in HBE cells, most notably CHIR99021 and dexamethasone. This effect was less pronounced when confirmed in non-COPD primary cells (Supplemental Figure 5). In an effort to identify possible disease-relevant upstream factors that may be a physiologic source for the PMA effect on *IL33* expression, we tested *Alternaria alternata* extract, protease activated receptor (PAR) agonist SLIGKV-NH2, and IL-4 in HBE cells. None of these stimulants increased *IL33* transcript expression (Supplemental Figure 5), suggesting that the relevant stimuli in vivo may be more complex and possibly involve multicellular or multi-hit mechanisms to drive IL-33 expression.

To test the effect of AP-1 and AP-2 TFs identified in the TF array, we conducted RNA interference experiments in HBEs (Figure 4C). Cells were transduced with lentiviruses expressing short hairpin RNA (shRNA) targeting AP-2 family members *TFAP2A* and *TFAP2C* and the required component of the AP-1 complex *JUN*. Expression of *IL33* transcripts was measured under the knockdown conditions and demonstrated a modest effect of *TFAP2A*, with a more marked effect with *TFAP2C* and *JUN*. The effect of *TFAP2A* knockdown appeared to be greater for *IL33A* than the noncanonical transcripts, suggesting that hetero- or homodimers of AP-2 TFs may differentially affect promoter activity. To confirm AP-1 regulation using a complimentary approach, a specific AP-1 inhibitor T-5224 was also tested that demonstrated significant reduction of IL-33 expression from all 4 promoters (Figure 4D and Supplemental Figure 5).

To determine whether PMA-induced IL-33 protein could also be secreted, ELISA was performed on cell supernatants and lysates following 12 hours of PMA treatment (Figure 4E). For TFAP2A knockdown, there was a moderate reduction in IL-33 protein, while *TFAP2C* and *JUN* both demonstrated a more pronounced effect on both secreted and total cellular protein. To confirm that secreted protein measured by ELISA was not due to cell death induced by PMA, lactate dehydrogenase (LDH) release assays were performed under knockdown conditions and demonstrated minimal cytotoxicity (Supplemental Figure 5).

We also analyzed PMA induction of IL-33 protein by Western blot under knockdown conditions. This revealed a similar pattern of reduced protein expression with knockdown of AP-2 and AP-1 TFs, and it included both an apparent ~34 kDa band corresponding to IL-33^full^ and with longer exposure a ~24 kDa lower band (Figure 4F). We attribute this lower band to protein expressed from a spliced isoform, most likely the exon 3–4 deletion variant (IL-33^Δ34^), as the protein was reactive only with a C-terminal domain (CTD) targeting antibody, not an N-terminal domain (NTD) targeting antibody, as previously described (25). Notably both the full-length and truncated bands show induction with PMA under control conditions, and this induction is diminished under knockdown conditions, most prominently for *JUN*.

These results show that PMA reproducibly induces IL-33 in HBEs through a mechanism that involves coordination of AP-1 and AP-2 TFs to drive noncanonical promoters and associated transcripts. Furthermore, PMA induction of IL-33 cellular protein allows for secretion that is, in part, dependent on AP TF activity. Western blot analysis comparing NTD and CTD reactive antibodies suggest that secretion could be related to increased production of IL-33^Δ34^ protein, which we have previously demonstrated is tonically secreted from airway cells through an extracellular vesicle–mediated (EV-mediated) pathway (25). Measurement of LDH release did not demonstrate substantial cellular toxicity under assay conditions, supporting a regulated secretion mechanism.

### Epigenetic regulation of AP-mediated IL-33 expression.

To further investigate how AP TFs regulate multiple promoters across the ~42 kb *IL33* gene locus, we took advantage of the observation that HBE, B2B, and 16HBE cell lines exhibit marked baseline differences in *IL33* expression from canonical and noncanonical promoters (Figure 3A). Because both AP-1 (33, 34) and AP-2 (35) TFs can be activated by phorbol esters, and AP-2 can function as a pioneer TF to regulate stem cell associated gene programs (36, 37), we examined *IL33* expression in the context of multiple AP-1 and AP-2 responsive promoters and/or enhancers within the gene locus.

**Figure 5:**
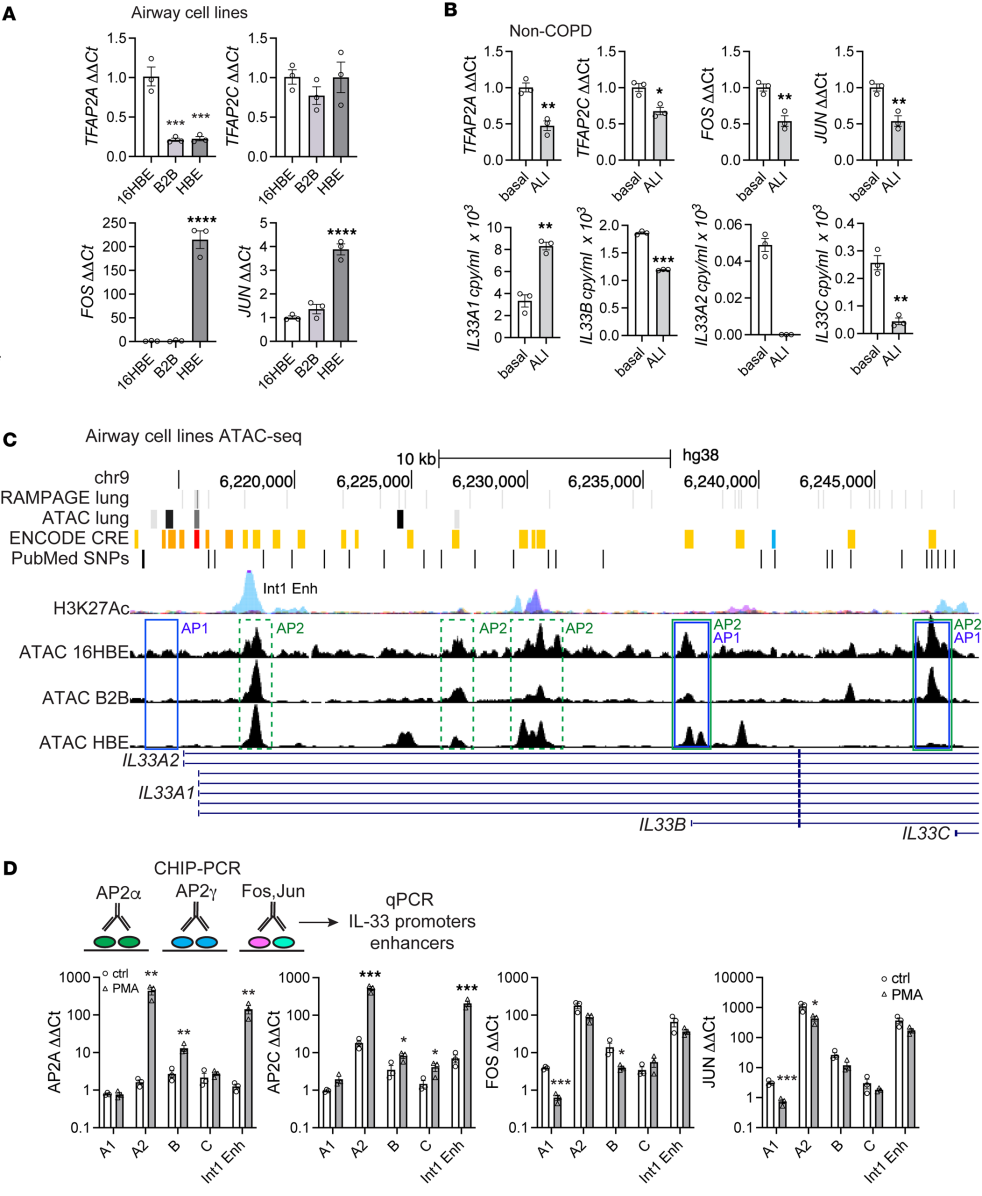
Epigenetic modulation of expression from *IL33* locus. (**A**) Expression levels of AP-2 (*TFAP2A*, *TFAP2C*) and AP-1 (*FOS*, *JUN*) transcription factors measured in HBE, B2B, and 16HBE cell lines. (**B**) Expression analysis for AP-1 and AP-2 TFs and *IL33* promoter–driven isoforms in undifferentiated primary airway basal cells and after 3 weeks in air-liquid interface (ALI) conditions, performed on the same non-COPD specimen. (**C**) ATAC-Seq performed on airway cell lines with chromatin-accessible peaks superimposed on the *IL33* promoters region, also mapped for comparison: whole-lung ATAC-Seq, RAMPAGE (promoter mapping), *cis*-regulatory elements (CRE), and H3K27 acetylation marks on human hg38. Promoter-derived transcripts are labeled. Predicted JASPAR transcription factor binding sites for AP-1 and AP-2 are labeled green and blue, respectively; dashed boxes represent putative enhancers and are color coded based on activator protein transcription factor sites. Summary of ClinVar SNPs is displayed as PubMed SNPs. (**D**) ChIP-PCR analysis performed for each of the *IL33* promoters and the labeled Intron 1 Enhancer (Int1 Enh). Immunoprecipitation was performed with AP-2α, AP-2γ, FOS, and JUN antibodies, as shown in the schematic. qPCR was performed with promoter-specific primer sets and reported as fold-enrichment (ΔΔCt) relative to bead-only control. Statistical analysis included 1-way ANOVA (**A**), *t* test (**B**), and multiple *t* test (**D**). *P < 0.05, **P < 0.01, ***P < 0.001, ****P < 0.0001. Experiments in **A** and **B** are representative of duplicate experiments, **C** is a single experiment, and **D** is representative of triplicate repeats.

First, we measured relative expression levels of AP-1 and AP-2 TFs in the cell lines under study and primary cells according to differentiation state (Figure 5A). Analysis of cell lines demonstrated similar levels of *TFAP2A* and *TFAP2C* expression, although with a significantly lower level for *TFAP2A* in HBE and B2B relative to 16HBE. AP-1 TFs can function as homo- or heterodimers, with JUN being the required subunit for activity (38). We examined both *FOS* and *JUN* expression patterns in the cell lines, and this revealed a very large differential expression for *FOS* in HBE relative to B2B and 16HBE and a less prominent though significant pattern observed for *JUN*. We then analyzed a non-COPD specimen cultured in a polarized format as undifferentiated basal cells or differentiated at air-liquid interface (ALI) for 3 weeks (Figure 5B). Under differentiated conditions, basal cells would be reduced in number, and bulk mRNA expression should reflect increased presence of ciliated, mucus, and secretory cell populations. Comparison of differentiation states revealed that *TFAP2A*, *TFAP2C*, and *JUN* were significantly decreased when cells were differentiated at ALI (Figure 5B). There was also a corresponding decrease in noncanonical *IL33* transcript expression, though canonical expression appeared preserved.

Together, these findings provide support for the role of AP-1 and AP-2 TFs in regulating *IL33* cellular expression, both in the high-baseline context of the HBE cell line and in basal progenitor cells. These results are consistent with prior reports implicating AP TFs in maintenance of stem cell programs and illuminate a potential basis for observed basal cell–restricted *IL33* expression in human airways.

To address the role of altered chromatin accessibility in AP TF activity and *IL33* expression patterns, we analyzed the 3 human cell airway cell lines with distinct baseline *IL33* expression levels by ATAC-Seq (Figure 5C). We identified multiple chromatin-accessible peaks within the *IL33* gene locus and compared them with whole-lung ATAC-Seq data and RNA Annotation and Mapping of Promoters for the Analysis of Gene Expression (RAMPAGE) data available through ENCODE (https://www.encodeproject.org; ref. 39), with custom tracks displayed on the genome in Figure 5C. Whole-lung ATAC-Seq performed on normal human specimens revealed prominent ATAC-Seq and RAMPAGE peaks within the *IL33A2* and *IL33A1* promoters, which may reflect a predilection for canonical *IL33* expression in multiple lung cell types including endothelial cells (40). In contrast, the HBE cell line exhibited prominent ATAC-Seq peaks corresponding to the noncanonical B promoter but not A2 or C promoters (highlighted by solid boxes), consistent with our noncanonical expression data for these transcripts. Of note, HBE cells still express *IL33A*, despite no prominent peak corresponding to the A1 promoter, as present in whole lung data. The B2B line demonstrated peaks only at the B and C promoters, while near-background peaks were observed across all promoters in 16HBE cells, consistent with undetectable expression in this cell line. In addition to core promoter regions, there are several putative enhancers throughout the *IL33* locus, highlighted by ENCODE *cis*-regulatory elements (CRE) and histone lysine acetylation (H3K27Ac) marks in Figure 5C. Multiple ATAC-Seq peaks are observed for the cell lines within the exon 1A1-1B intronic region corresponding to CRE and H3K27Ac marks (highlighted by dashed boxes; Figure 5C), which could function as enhancers and potentially explain the observed *IL33A1* expression in HBE cells. We also analyzed JASPAR-predicted (41) AP-1 and AP-2 TF binding sites in the *IL33* locus, which were located in multiple promoters and putative enhancer regions as highlighted in Figure 5C. The A2, B, and C promoters all contain a proximal AP-1 site with adjacent AP-2 sites for B and C, while A1 has neither of these sites in the core promoter region. Of note, there are additional AP-2 sites located in 3 putative enhancers within the exon 1A1-1B intron, which correspond to our ATAC-Seq peaks in HBE as well as CREs and H3K27 acetylation marks. The putative enhancer region closest to the A1 promoter has been designated Intron 1 Enhancer (Int1 Enh) in Figure 5, C and D.

Given the presence of *IL33A1* expression in HBE despite the absence of an ATAC-Seq peak in the promoter region, we performed ChIP-PCR analysis using unstimulated and PMA-stimulated HBE cells with IP for antibodies to AP-2α, AP-2γ, Fos, and Jun. Results are graphed as fold-change enrichment relative to bead control in Figure 5D. This analysis revealed that there was relatively low-level enrichment of the A1 promoter for AP-2 targets, but there was moderate baseline enrichment for AP-1 targets, which decreased in response to PMA. In contrast, the A2 promoter was enriched at baseline for AP-2 and AP-1 targets, with both Fos and Jun ChIP demonstrating a high level of baseline enrichment — 100- and 1,000-fold, respectively. In response to PMA treatment, AP-1 factors did not further enrich A2, but there was a dramatic effect for AP-2 TFs (100-fold increase), suggesting part of the PMA effect on *IL33* expression is mediated through the region of the A2 promoter. Likewise, AP-2 and AP-1 TFs mildly enriched the B promoter, which was augmented by PMA for AP-2 TFs. The pattern observed for the B promoter was mirrored at the C promoter, with mild enrichment for AP-2 TFs with PMA. Interestingly, analysis of the first putative Int1 Enh located ~1 kb distal to the A1 promoter demonstrated high level enrichment with AP-1 targets at baseline and a strong induction of AP-2 enrichment with PMA. This result, in combination with observations for the A2 promoter, suggests that AP TFs could modulate *IL33A1* and *IL33B* expression through nearby enhancers.

Multiple prior reports have implicated SNPs within the *IL33* locus with disease-associated changes in *IL33* expression (42–44). Based on our results above, we cataloged SNPs reported in PubMed from ClinVar database (https://www.ncbi.nlm.nih.gov/clinvar) that were inclusive of the *IL33* promoter and enhancer regions (chr9: 6193455–6248408) and overlayed on our ATAC-Seq data in Figure 5C. While none of these SNPs localized to the core *IL33A1*, *IL33A2*, or *IL33B* promoter regions, some overlapped with ATAC-Seq peaks in the C promoter region (rs79981454, rs7037276, rs10975516, rs11792633, and rs76864631). Some clinically relevant SNPs were also located approximately 1,500 bp proximal to the A2 promoter (rs928413 and rs7848215) and others 400 bp distal to the A1 promoter (rs1157505 and rs11791561), but none of these corresponded to ATAC-Seq peaks in our data set. Multiple SNPs were located within ATAC-Seq peaks in the 1A1-1B intron region that correspond to putative enhancers (dashed boxes, including rs72614080, rs16924159, rs16924161, and rs12551256). Among these, rs72614080 is associated with prognosis in osteosarcoma (45), rs16924159 with vascular disease (46), and an off-peak SNP rs1891385 with COPD (47). Other SNPs associated with airway disease (42) are located ~6 kb proximal to the A2 promoter (rs992969 and rs3939286) and do not correspond to ATAC-Seq peaks observed in this data set; however, an AP-1 binding site was reported within the region encompassing these SNPs.

These data indicate that *IL33* expression from canonical and noncanonical promoters is coordinated by interaction of AP-1 and AP-2 TFs with *IL33* promoters and enhancer regions. This regulatory program appears to be active in airway basal progenitors and may be lost as cells become differentiated. Expression from multiple noncanonical *IL33* promoters can be partly explained by differences in chromatin accessibility at core promoters and may also include long-range effects mediated through enhancers in the 1A1-1B intron. Polymorphisms within the *IL33* locus have been studied; however, based on the ATAC-Seq data from airway cell lines, these do not correspond to peaks within *IL33* core promoter regions. Instead, they appear to be associated with enhancer regions, as an AP-1 site is located in an upstream regulatory region and a SNP associated with COPD is located near the 1A1-1B intronic enhancer region. There also remains potential for numerous uncharacterized SNPs within *IL33* enhancers to regulate expression from canonical or noncanonical promoters in airway cells.

### AP TFs are increased in COPD.

**Figure 6:**
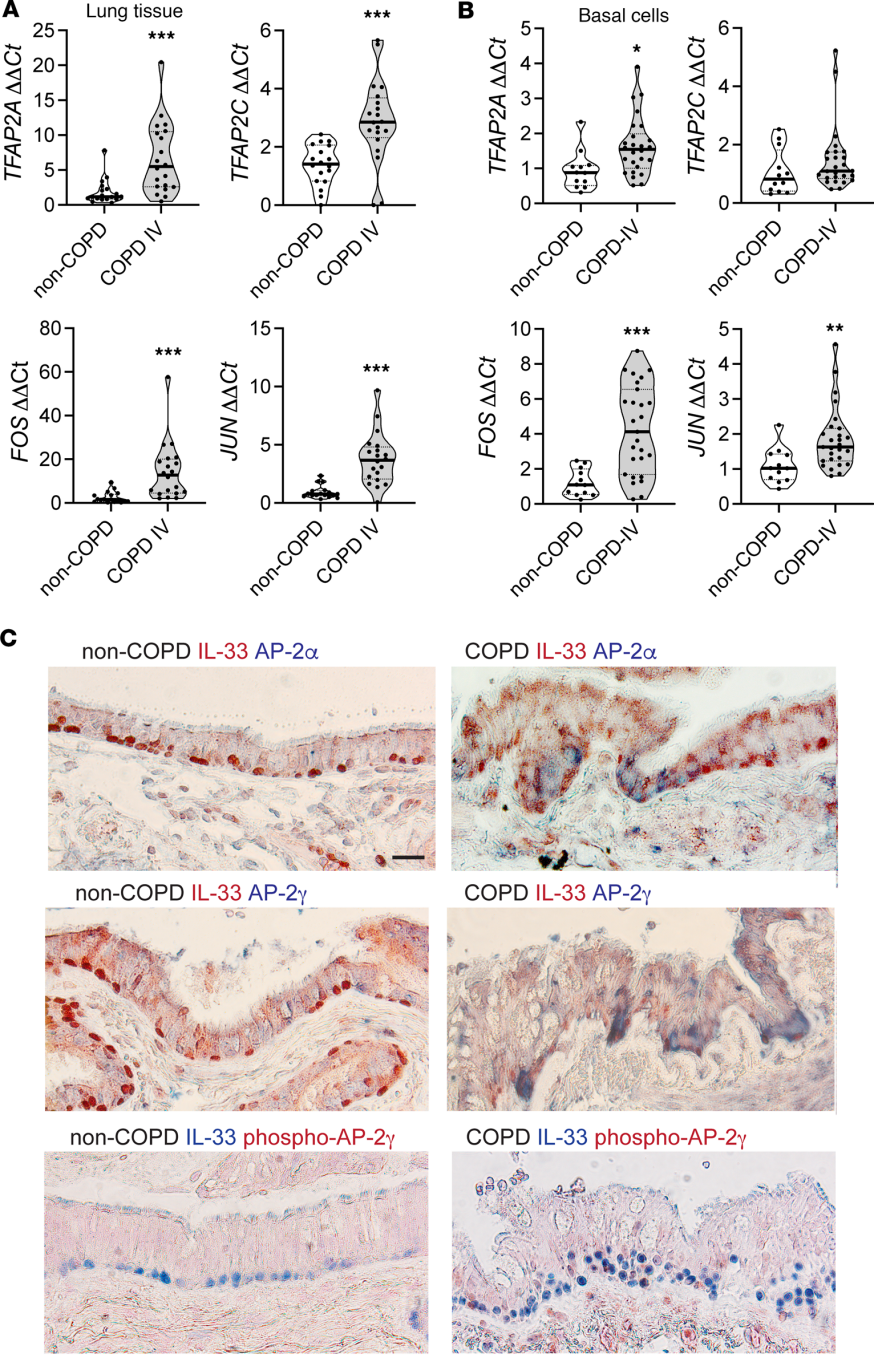
AP transcription factors in COPD. (**A**) *TFAP2A*, *TFAP2C*, *FOS*, and *JUN* expression in COPD lung tissue demonstrates significantly increased expression for all transcription factors in COPD relative to non-COPD, represented as fold change ΔΔCt normalized to *GAPDH*. Cohort includes *n* = 18 non-COPD and *n* = 20 COPD specimens; each data point represents an average measurement from 4 different lung regions. (**B**) Analogous expression levels in airway basal cells for *n* = 12 non-COPD and *n* = 25 COPD specimens. (**C**) IHC staining of non-COPD and COPD lungs for IL-33 costaining with AP-2α or AP-2γ demonstrates enrichment of TF signal in COPD airways. Costaining of IL-33 and phospho–AP-2γ demonstrates enrichment of nuclear AP-2γ signal in COPD tissue. Images taken with 40× magnification. Scale bar: 10 mm. Statistical analysis included *t* test (**A** and **B**). *P < 0.05, **P < 0.01, ***P < 0.001. Experiments in **A** and **B** are representative of duplicate technical replicates; **C** staining was repeated in triplicate.

Given our findings that AP-1 and AP-2 TFs regulate canonical and noncanonical *IL33* promoters, we examined TF expression patterns in COPD and non-COPD lung tissue. On the mRNA level, all 4 AP-1 and AP-2 TFs examined were significantly increased in COPD relative to non-COPD control lung tissue (Figure 6A). A similar pattern was observed in primary airway basal cells, but only the AP-2 TF *TFAP2A* was significantly increased in COPD compared with non-COPD cells (Figure 6B). Both *FOS* and *JUN* were markedly and significantly upregulated in lung tissue and airway cells. On the protein level, tissue immunostaining demonstrated that AP-2α and AP-2γ were both enriched within COPD airways and staining colocalized at the base of the epithelium within IL-33^+^ basal cells (Figure 6C). To evaluate potential AP-2γ activation in lung tissue, sections were stained for phosphorylated protein, which revealed focal nuclear staining in IL-33^+^ basal cells. These data support a role for increased AP-1 and AP-2 TF activity in COPD disease, which we have shown functionally induces *IL33* expression from both canonical and noncanonical promoters that influence alternative splicing patterns.

## Discussion

Our study has uncovered a potentially novel pathway for amplification of IL-33 cytokine expression by AP TFs in chronic airway disease. We observe that IL-33 can be expressed from noncanonical promoters, resulting in unique transcripts with increased propensity for alternative splicing and cytokine secretion, a phenomenon that appears active in COPD. We found that noncanonical *IL33* promoter induction was responsive to phorbol esters and occurred through a PKC-dependent pathway. We further demonstrate epigenetic regulation of the *IL33* locus through chromatin accessibility of promoters and enhancers that are integrated by AP-1 and AP-2 TFs in epithelial progenitor cells. A similar phorbol ester and PKC-dependent axis has been observed in primed T cells that was shown to induce IL-13 secretion (48). In that study, AP-2 was found to be competitive in PMA-responsive EMSA assays. Together, these studies suggest that PKC- and AP-1/AP-2–mediated cytokine regulation may represent a unifying mechanism of promoting type 2 responses in multiple cell types within the respiratory mucosal interface, with relevance to airway disease.

What remain unknown are the airway disease–related upstream genetic, epigenomic, or environmental factors that give rise to this PMA-inducible phenomenon. A possible mechanism for epigenetic modification of the *IL33* locus may be through altered histone deacetylase activity, as has been observed in multiple inflammatory diseases including COPD (49, 50). Likewise, uncharacterized SNPs within promoter and enhancer regions need further exploration, such as SNPs within or near the Int1 Enh region we have highlighted here. Another consideration is sex-specific differences in *IL33* promoter regulation. We observed that the A1 promoter strongly competed with ER binding in cells derived from a female donor. While we could not confirm ER expression in the HBE cells, it is possible that related pathways could promote sex hormone–mediated expression of *IL33* through the A1 promoter. Increased incidence of asthma after puberty is well described in women (51), and AP-2 has been shown to mediate long-range effects on gene transcription via the ER (52), so this potential mechanism warrants investigation in future studies. Last, there are several potential mediators that could signal through GPCRs to activate G-protein signaling and diacylglycerol production, a process that phorbol esters mimic (53). Among these mediators could be endogenous-derived signaling chemicals or lipids that are liberated in response to allergen exposure, infection, or tissue damage in order to integrate extracellular signals into a stereotypical response to environmental cues. Identification of these triggers will be the focus of future studies.

We found that differences in chromatin accessibility were associated with distinct *IL33* expression patterns in airway cell lines; however, none of the cell lines we examined harbored peaks at the canonical *IL33A1* or *IL33A2* promoters. Comparison with whole lung ATAC-Seq and RAMPAGE data demonstrates that A1 is likely the dominant promoter on the whole lung level, possibly reflecting contributions from other cell types that express IL-33, including endothelial cells and fibroblasts (22). Chromatin accessibility of the A1 promoter could very well be a mechanism for regulating baseline versus inducible or responsive expression in distinct cell types, possibly with enhancer-mediated effects playing a dominant role in airway cells. It will be of great interest to compare the relative chromatin accessibility of *IL33* promoters in other lung cell types, which will provide insight into how chronic airway disease is initiated and propagated by environmental triggers through cell-specific expression characteristics.

The role for AP-1 and AP-2 TFs in regulating a transcript associated with epithelial progenitors may reflect the reported function of AP-2γ as a pioneer TF in stem cell and differentiation programs (36, 52). It is conceivable that this TF could function to maintain noncanonical *IL33* promoters in open conformation in stem cells of barrier tissues in order to rapidly respond to a variety of danger signals, a pathway that may be further enriched in disease. It will be of interest in future studies to determine whether increased AP-1 and/or AP-2 activity mediated by phorbol esters licenses *IL33* promoters to respond to environmental triggers differentially or whether this represents a steady-state expression phenomenon. To fully explore this will require a similar analysis as presented here for multiple distinct cell types with low- and high-baseline IL-33 expression and the identification of endogenous stimuli that drive this program.

In summary, we previously described how increased expression of a truncated *IL33^Δ34^* isoform with altered cellular distribution resulted in tonic secretion from airway basal cells by coopting the EV biogenesis machinery (25). In this study, we have begun to unravel the mechanism that gives rise to alternate forms of IL-33 and how expression can be induced in chronic airway disease. Subsequent work will seek to increase mechanistic understanding of cell type–specific *IL33* transcriptional control, regulated secretion intermediates in respiratory mucosa, and cell-specific downstream signaling networks to develop precision targets and therapeutics for type 2–driven chronic airway disease.

## Methods

### Sex as a biological variable.

Our study examined similar numbers of male and female human lung specimens in COPD and non-COPD cohorts, and they were obtained as available based on candidacy for lung transplantation and consent for study. Similar findings are observed for both sexes.

### Human lung samples and study design.

Clinical samples were obtained from consenting patients at the time of lung transplantation from COPD recipients (*n* = 20) with very severe disease (GOLD Stage IV) during the period from 2016 to 2022 at Barnes-Jewish Hospital (BJH, St. Louis, Missouri, USA). Control samples were obtained from non-COPD donor lungs (*n* = 18) that were not useable for transplantation under a separate approved protocol. For COPD specimens, there were no predetermined inclusion or exclusion criteria beyond criteria for lung transplant candidacy. For non-COPD specimens, exclusion criteria included pneumonia as defined by consolidation on CT imaging and/or positive respiratory culture. To analyze tissue staining and gene expression, lung tissue samples were collected and processed for histopathology and RNA analysis from 4 different lung zones of each specimen. For this study, equivalent quantities of lung tissue were homogenized in Trizol (Invitrogen), and an aliquot from each of the 4 different lung areas was processed for RNA analysis, with a single pooled representative sample per specimen. Tissue specimens were fixed in 10% neutral buffered formalin (Thermo Fisher Scientific) prior to paraffin embedding and sectioning for histopathology analysis. Airway basal cells were dissociated from large airways (first to third generation) using 0.15% Pronase (Roche), cultured in serum-free bronchial epithelial growth medium (BEGM) and analyzed at low passage. Cells were processed for RNA extraction using Trizol, as above.

### qPCR assays.

To quantify alternate-promoter *IL33* transcripts, we designed a series of qPCR assays to probe the unique 5′UTR exons 1A2, 1A1, B, and C. A specific assay could not be generated for *IL33A1* based on the cloned *IL33A2* transcript (Supplemental Figure 2). For qPCR analysis, total RNA was purified from lung homogenates and cell lysates using Trizol (Invitrogen) extraction and converted to cDNA template using a High-Capacity cDNA Archive kit (Applied Biosystems) or Ambion cells-to-cDNA II kit (expression screen), both according to manufacturer protocols. Target mRNA expression was quantified by qPCR assay fluorogenic probe-primer sets (labeled with 5′FAM and 3′-IowaBlack), either designed using the PrimerQuest tool through the Integrated DNA Technologies (IDT) website (https://www.idtdna.com; *IL33* isoform-specific assays) or using IDT predesigned validated assays for other targets. qPCR was performed using the KAPA PCR Master Mix system (KAPA Biosystems). Samples were assayed with the 7500 Fast Real-Time PCR System and analyzed using Fast System Software (Applied Biosystems). Transcript copy/mL was quantified based on Ct for human *IL33* transcripts using plasmid standards for cloned transcripts. For targets without available plasmid standard, fold-change was calculated using the ΔΔCt method (relative to average for control group). Samples that did not amplify with a Ct value threshold < 35 for a given target were reported as not detected. In all cases values were normalized to *GAPDH* (Applied Biosystems human and human GAPDH 20X assays).

### Cloning and construct generation.

For cloning of *IL33* isoforms from COPD airway basal cells, PCR was performed on cDNA template using primers within the unique exon 1 regions or the coding region encompassed by exons 2–8, using Pfu UltraII High Fidelity Master Mix (Agilent) according to manufacturer protocol. PCR products were separated on 1% agarose gel and visualized with ethidium bromide; images were captured on a Thermo iBright 1500 platform. PCR bands amplified with IL-33 coding sequence primers (exon 2 and 8 with NdeI and XhoI restriction sites) were extracted using Qiagen gel extraction kit and were Sanger sequenced. PCR products amplified with unique exon 1 were digested with restriction enzymes above, ligated into pET23b expression vector, and transformed into NEB 10-beta *E. coli* for plasmid purification (New England Biolabs). In the case of *IL33C*, a construct was generated using IDT gBlocks and Gibson assembly (New England Biolabs) according to manufacturer protocol. These plasmids were used as standards for qPCR.

### Airway epithelial cell culture.

Primary culture airway basal cells were established from large airway (first to third generation) tracheobronchial specimens as described (54, 55); they were cultured in 2D submerged format on collagen-coated plastic or transwell inserts (Corning) and maintained at 37°C 5% CO_2_ in BEGM without serum (56). HBE and B2B cell lines were grown in BEGM, and 16HBE cell lines were grown in α-MEM–based medium with 10% serum according to manufacturer protocol (MilliporeSigma). For experiments, cells were seeded at 20,000 cells/well in a 96-well format and grown to 70% confluence prior to study. In some experiments, airway cells were differentiated at ALI using standard protocols (54). Airway cells were lysed either in Trizol or SDS-PAGE sample loading buffer for mRNA or protein analysis.

### IL-33 secretion ELISA.

Cells were plated in a 96-well format and treated with PMA for 12 hours before incubation followed by a media change for a 2-hour secretion assay. Supernatant was collected, and cells were lysed in MPER (Pierce) with HALT. Supernatant and lysate IL-33 levels were measured using a human IL-33 ELISA DuoSetâ (R&D Systems) according to the manufacturer’s protocol. Chromogenic signal was developed using TMB substrate (SeraCare), and absorbance at 450 nm was measured on a BioTek Synergy H1 system.

### ChIP-PCR.

ChIP-PCR was performed using the Pierce Agarose ChIP Kit by Thermo Fisher Scientific (catalog 26156) following manufacturer protocol. Briefly, HBE cells were cultured in 15 cm tissue culture plates under submerged conditions until 70% confluence; some cells were treated with PMA (6 hours) prior to ChIP-PCR protocol. Cells were fixed with 1% paraformaldehyde for 10 minutes while shaking, followed by glycine for 5 minutes. Cells were then washed with PBS, scraped, and collected in PBS/HALT cocktail. The concentration of micrococcal nuclease was experimentally determined to optimize fragment size (~600 bp), and chromatin was digested at 37°C for 15 minutes. Nuclei were recovered by centrifugation at 9,000*g* for 5 minutes at 4°C, and a fraction was saved as the input sample. The remaining solution was diluted 10× in IP dilution buffer and incubated with anti–AP-2α, anti–AP-2γ, anti-FOS, or anti-JUN antibodies (1 µg) or bead control (see Supplemental Table 2 for antibody information). Lysates were incubated at 4°C overnight then incubated with ProteinA/G agarose for 1 hour at 4°C. Washed resin was resuspended in provided IP Elution buffer and incubated at 65°C for 30 minutes. Eluted and input samples were treated with proteinase K and RNase A (Qiagen) for 1.5 hours at 65°C. DNA was recovered using clean-up kit. qPCR was performed using SYBR Select Master Mix (Applied Biosystems) and primer probes for the promoter sequences A1, A2, B, and C, as well the Int1 Enh (Supplemental Table 2). qPCR was performed with the 7500 Fast Real-Time PCR System. Fold-enrichment relative to bead-only control was performed using the ΔΔCt method.

### Tissue IHC.

Tissues were fixed with 10% neutral buffered formalin, embedded in paraffin, cut into 5 μm sections, and adhered to charged slides. Sections were deparaffinized in Fisherbrand CitroSolv, rehydrated, and treated with heat-activated Vector Citrate antigen unmasking solution (Vector Laboratories). After blocking with BLOXALL Endogenous Blocking Solution (Vector Laboratories), followed by Tris buffered saline with 0.1% Triton X100 and animal free blocking solution, samples were incubated at 4°C overnight or for up to 48 hours using primary antibodies at dilutions outlined in Supplemental Table 2. After washing and incubating with horseradish peroxidase– and/or alkaline phosphatase–labeled secondary antibodies using ImmPACT HRP and AP kits (Vector Laboratories) IHC signal was developed with AMEC Red, ImmPACT NovaRED, or Vector Blue substrates (Vector Laboratories). Tissues were counterstained with nuclear fast red (Vector Laboratories). Bright-field imaging was performed on an Olympus IX83 inverted microscope with 40× and 60× objectives and a bright-field Olympus SC50 camera.

### TF binding array.

HBE-1 cells were grown to 70%–90% confluence on 150 mm tissue culture plates, and cells were subsequently lysed. The nuclear extract was obtained using a Nuclear Extraction Kit (Signosis) according to manufacturer protocol. The Promoter-Binding TF Profiling Array I (Signosis) was performed with arrays containing 10 µg A1, A2, or B promoter PCR products or no-input control, and 10 μg HBE-1 nuclear extract was mixed with biotin-labeled oligos corresponding to 48 TF binding sequences. The bound probes were separated from the free probes through column extraction. The bound probes were then hybridized overnight on a 96-well plate, and the signal was detected using Streptavidin-HRP and chemiluminescent substrate with luminescence measured on a BioTek Synergy H1 System. Percent inhibition of luminescence signal calculated relative to no competitive DNA input control was reported.

### Western blot.

For analysis of IL-33 proteins in cell samples, gel electrophoresis was performed using NuPAGE 4%–12% Bis-Tris Protein Gels (Invitrogen), followed by transfer to nitrocellulose membrane using iBlot 2 system (Invitrogen). Membranes were washed in PBS 0.1% Tween-20 (PBST), blocked in PBST with 2% BSA, and incubated with anti–IL-33 (goat, R&D systems) or anti–IL-33 propeptide (goat, R&D systems) at dilutions specified in Supplemental Table 2 while rocking overnight at 4°C. Membrane was washed and incubated with 1:5,000 dilution of anti-goat HRP–conjugated secondary antibody (R&D Systems) at room temperature. Western blots were developed using ECL Substrate (Pierce), and chemiluminescence was detected and band intensity was quantified using an iBright 1500 imaging system.

### Lentiviral shRNA knockdown.

For shRNA assays, low-passage HBE cells were trypsinized and plated with high-titer shRNA lentiviruses purchased from Santa Cruz Biotechnology Inc. and with protamine at 1 μg/mL. After 24 hours, virus was removed and media were replaced with BEGM containing puromycin at 5 μg/mL. Media were changed daily until cells had visibly recovered to 50%–70% confluence. Cells were used in secretion and Western blot assays or were lysed in Trizol, and cDNA was prepared to verify knockdown by qPCR and to measure expression of *IL33* isoforms.

### ATAC-Seq.

ATAC-Seq was performed on the 3 airway epithelial cell lines HBE, B2B, and 16HBE. Standard nuclear isolation, Illumina library generation, and transposition reaction methods were with input 1 × 10^6^ cultured cells. Quality of input material was assessed by Bioanalyzer tracing prior to proceeding with Illumina sequencing at 2 × 150 bp (350 million reads). ATAC-Seq data for all the cell lines were separately processed by the AIAP package that contained an optimized ATAC-Seq data QC and analysis pipeline with default parameters (57), including prealignment QC (FastQC), prealignment data processing (cutadapt), alignment to human reference genome hg38 (bwa), postalignment QC, and peak-calling by using MACS2 to identify open chromatin regions (OCRs). AIAP calls FastQC to check the sequencing quality, duplication rate, and GC bias before alignment. After alignment, AIAP generates the mapping statistics summary, chromosome distribution of uniquely mapped reads, mitochondrial genome (chrM) contamination rate, library insert fragment size distribution, library complexity, and ENCODE blacklisted regions removal. AIAP also performs a series of post–peak calling quality checks, including peak width distribution, reads under peak ratio (RUPr), background (BG), promoter enrichment (ProEN), subsampling enrichment (SubEn), saturation analysis, promoter peak distribution, and signal ranking analysis. OCRs and normalized ATAC-Seq signal visualization generated by AIAP was uploaded to the UCSC genome browser. Human lung RAMPAGE (ENCBS711XVO) and ATAC-Seq (ENCSR647AOY) data were downloaded from the ENCODE data portal (https://www.encodeproject.org/) and displayed with cell line ATAC-Seq data. SNPs accessioned in PubMed were accessed via ClinVar database (https://www.ncbi.nlm.nih.gov/clinvar/); JASPAR-predicted (41) TF binding sites within the *IL33* promoters region were added to custom tracks using the genome browser. All custom tracks were included in the graphical representation of the *IL33* gene locus shown in Figure 5C.

### Statistics.

For statistical analysis, 2-tailed Student’s *t* test was used for comparisons between 2 groups, and comparisons with 3 or more groups were analyzed using 1-way ANOVA. For all experiments, *P* < 0.05 was considered statistically significant. Correlation analysis was performed based on Pearson’s coefficient. For all data in which 3 or more independent measurements are reported, data are displayed as mean ± SEM.

### Study approval.

All human studies were conducted with protocols approved by the Washington University IRB, and written informed consent was obtained from study participants.

### Data availability.

All sequencing data in this paper have been deposited through Gene Expression Omnibus (GEO) repository (accession no. GSE249720). Values for all data points in graphs are reported in the Supporting Data Values file.

## Author contributions

HER, GFH, LSC, EKK, JH, OAO, IT, and JAB designed and performed the experiments; HER, GFH, and JAB prepared figures and wrote the manuscript; MDP edited the manuscript and generated the graphical abstract, BZ analyzed and interpreted ATAC-Seq data; DEB contributed IRB protocol management and human specimens biobanking. HER performed most experiments for the first submission; GFH performed experiments, analyzed data, prepared figures, and edited the manuscript for the first submission and performed all experiments and figure and manuscript revisions for subsequent submissions.

## Supplementary Material

Supplemental data

Unedited blot and gel images

Supporting data values

## Version 1 03/08/2024

Electronic publication
