# Supplementary material for: Activator protein transcription factors coordinate human IL-33 expression from noncanonical promoters in chronic airway disease
Source: JCI Insight. 2024 Mar 8;9(5):e174786. doi: 10.1172/jci.insight.174786 (PMC10972587; doi:10.1172/jci.insight.174786)
Supplement: Supplemental data [file jciinsight-9-174786-s206.pdf]

## SUPPLEMENTARY MATERIALS

**TITLE: Activator protein transcription factors coordinate human IL-33 expression from non-canonical promoters in chronic airway disease**

**Supplementary Figures.**

**Supplementary Figure 1.** Cloned *IL33* transcript sequences associated with Figure 1.

**Supplementary Figure 2.** Validation of isoform-specific assays, associated with Figures 1 and 2.

**Supplementary Figure 3.** Jurkat PMA expression data and B2B PKC inhibitor data associated with Figure 3.

**Supplementary Figure 4.** Single-cell TF expression bubble plots generated from LungMap data, associated with Figure 4.

**Supplementary Figure 5.** HBE and basal cell response to TF activators, associated with Figure 4.

**Supplementary Tables.**

**Supplementary Table 1.** Clinical characteristics of subjects without COPD (Non-COPD) and with severe (GOLD Stage IV) COPD, related to Figure 1.

**Supplementary Table 2.** List of reagents and materials.

**Supplementary Table 3.** List of reagents and concentrations used for IL-33 expression screen.

**Supplementary Table 4.** List of reagents and concentrations used for expression screens in follow up to TF binding array.

## SUPPLEMENTARY FIGURES

IL33A2 (new transcript) promoter region, [exon 1A2'], exon 1A2, exon 1A1 and exon 2

CCCCTCCCTCCTATTTATTGCACACAGCTCAATTCCTGATTCATTCTTGTCATGTTGGCATCCT
GTCCGCTTGCTTCACTGCTTTATCCAAAGGGCAGACATAGCCATCTTTCTTCATTACTCACAAA
TGCAACACTATGGCTTGTAGCAGATGATTGCTCAGGTTTGGGGATGATTAAAGGGGATTACTGC
ACAGAAAATGAAACAACTTTAAACAAGATAACAGAGTTCCTTAGTTGTTTTTTCCAAAGGGGAC
TCCATCATAGTCACATTTTCCCAAGCCTGGTCAGCCATCTGTTGCTGTCTCATTTTCTCTTTCC
CCTGGATTCCAGAACCCTAACATTCTATCCTTCAACATGCCATGAGAAAAACTAAAAAATTCCT
ATTTCATTTTACAGTAAACTTTTTTCTGCTGCCCCTTTGTTTCAAGGTCACCCTTCTAATCTTG
TAGATTGAATGGATGTAGAGGCTTGCTAGGGTAACAGTCCATCACAATATCCTTCTAAGTACAG
CAGCAGTCTTCCTTCCAATGTGTGATCTTAGGATGTGGAATCAGTCCAAGTCT**TAAAA**GAGCAT
TACTTCATCCCTTTATTTTCTGCT[ACAGATGCCAAACGAGATGGAGAGAGG]ATCTCCCTTCT
AAGGCAATTTGGGTCTCTGCCAAACTTTGGCTAATAAAAAGAGTCTACAGACTCCTCCGAACAC
AGAGCTGCAGCTCTTCAGGGAAGAAATCAAAACAAGATCACAAGAATACTGAAAAATGAAGCCT
AAAATGAAGTATTCAACCAACAAAATTTCCACAGCAAAGTGGAAGAACACAGCAAGCAAAGCCT
TGTGTTTCAAGCTGGGA

IL33A1 (NM_033439.4) promoter region, exon 1A1 and exon 2

GCAAAATTTCTCATGAGGTAAGAAGAACAACTTCTCATGGGATCTATAGTACAGGATTTGCTTT
TGTGATAAAGTATTATCTTCTGCGAGATTTTTGAGATGAATAGAAAGAAAAGATGAACAGTGGG
GAGCAGGAAAGCCCGTCAGATATGTTGGAATACAGAGTATTTCAGAGCTTTTATTTGCAGACGA
GCTGCTTATTACTGATAGGCCAAAGGGGGTCGCAAATTTCTATTATAAAGGAATTTGGAAGAAT
ACACTGATCTCTTAATGAAGTTTGTAAATTAAGAAGCCAACGGCCAAAAGTGAATATATAAATG
GCAACAGAATTTCAAAATGGACAGTGCTGAAGTTGAAGTTTAGGAGTTCAACTCCAAAGAGAGC
CAAAACATAAAGTTTAGGGGCAGAAGAAGAATCATAATTGCTGGTTTAAAATATTCAGATGGAG
GGAGGACGCAGAAAGTAGTGAGCCTTAGATGTTGACAGAATTGTAACTCTGTTGGCTCTTTACA
TGAGATTTCAAGCCTGCTAAAATCTCACCCGCCCAGATCTCCCTTCTAAGGCAATTTGGGTCTC
TGCCAAACTTTGGC**TAATAA**AAAGAGTCTACAGACTCCTCCGAACACAGAGCTGCAGCTCTTCA
GGGAAGAAATCAAAACAAGATCACAAGAATACTGAAAAATGAAGCCTAAAATGAAGTATTCAAC
CAACAAAATTTCCACAGCAAAGTGGAAGAACACAGCAAGCAAAGCCTTGTGTTTCAAGCTGGGA

IL33B (NM_001314045.2) promoter region, exon 1B and exon 2

CCTTCTATATTATATGTTATGCAATAAACACTGCCTAGAGCCTATATTGCAAAGATTTTATAAA
AGTTAATGATTAACTAAGTGTAACTCTTTGACAGTGCTACAACTAGGCCGGGTGCAGTGGCTCA
CGCCTGTAATCCCAGCACTTTGGGAGGCTGAGGCAGGAGGATCACTTGCGGTCAGGAGTTCAGG
ACCAGCCTGGCCAACATGGTGAAACCCCATCTCTATAAAAATACAAAAAAAATTGGCCAGGTGT
GGTGGCATACACCTTGTAGTCCCAGCTACTTGGGAGGCTGAGACAGAAGAATTGCTTTAATCTG
GGAGGTGGAGGCTGCAGTGAGCTGAGATCGTGCCACTGCACTCCAGCCTGGGCAGCAGAGGGAG
ACTCTGTCTCAAAAAATAAATAAATAAATAAAATGAAAGTGCTACAGCTAAAATAATCTCTGTT
TAGGATCTGACGGTAGGAAAAAGATAAGCTCCCACACGTTCTAATGCATTTAAGTAGCTCCATC
TGCATTGCCTCATACATCAATACTGTCATCCAATGTTGCTTAATCCATGTGTGTGTGTGTCTA**T**
**ATAA**GTAGAACTGGGATGTAACTGCCTTAAATACTACAATTGCTGACTACAGGAAACCTCATCA
TCTGAGACCAGCACTTTATAAATTAGAATACTGAAAAATGAAGCCTAAAATGAAGTATTCAACC
AACAAAATTTCCACAGCAAAGTGGAAGAACACAGCAAGCAAAGCCTTGTGTTTCAAGCTGGGA

**Supplementary Figure 1. Cloned *IL33* transcript sequences.** Core promoter sequences used in transcription factor binding array with black text, confirmed by Sanger sequencing. 5’ UTR exon 1A1, 1A2-1A1, and 1B are shown based on cloning from COPD airway cells.

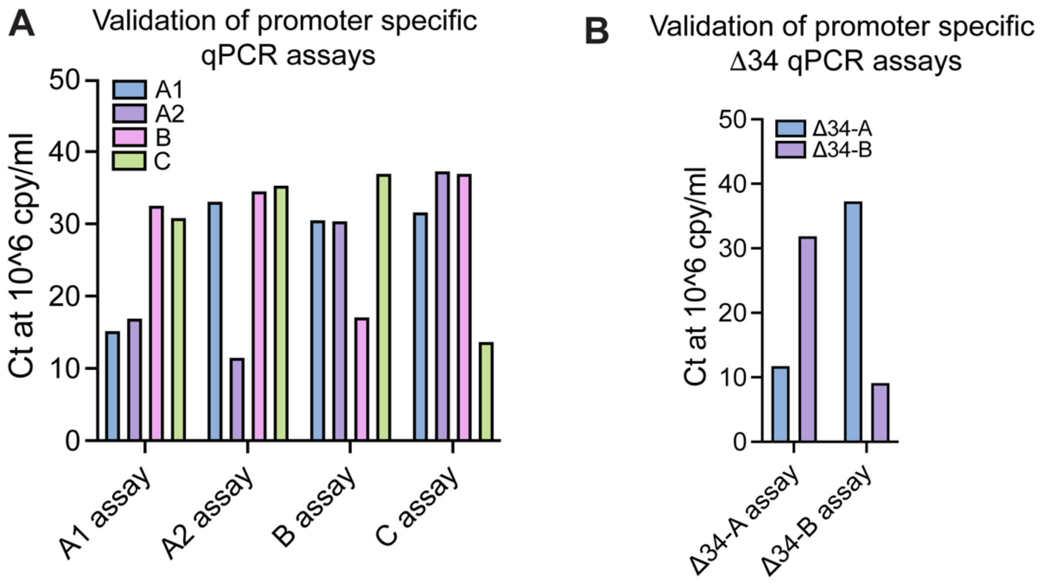


**Supplementary Figure 2. Validation of isoform-specific assays. A)** Cycling threshold (Ct) at 10^6 cpy/ml using plasmid standards for promoter-specific transcripts demonstrating specificity for *IL33B*, *IL33C*, and *IL33A2* assays, but amplification on both *IL33A1* and *IL33A2* for the *IL33A1* assay. **B)** Comparative expression at 10^6 cpy/ml performed using plasmid standards for $IL33^{\Delta 34}$ based on promoter specific transcripts for *IL33A1*, *IL33B*. Experiments are representative of duplicate repeats.

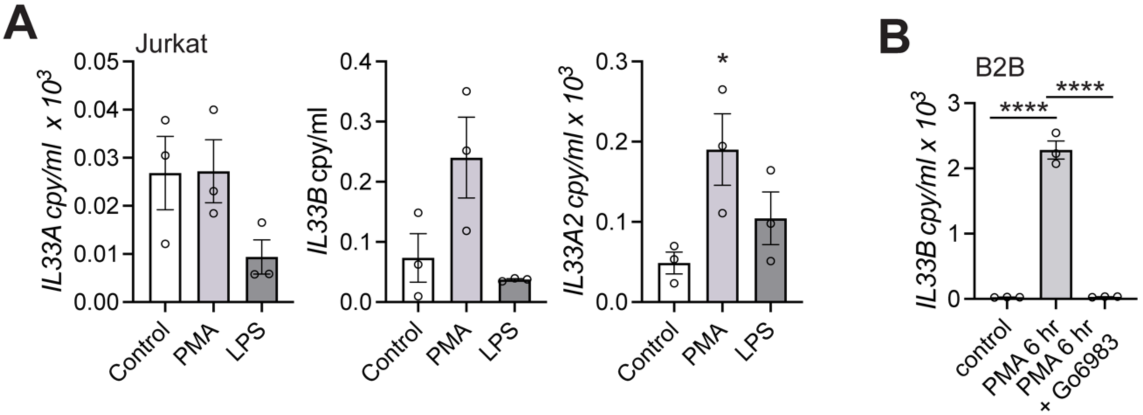


**Supplementary Figure 3. Jurkat PMA expression data and B2B PKC inhibitor data.**

**A)** Jurkat expression in response to PMA measured for *IL33A*, *IL33B*, and *IL33A2* assays, *IL33C* was not detected. **B)** PMA induction of *IL33B* in B2B cells treated with or without the PKC inhibitor Go6983, other *IL33* promoter-specific isoforms were not detected. Statistical analysis: one-way ANOVA (A, B), *P*-value limits: $*P < 0.05$, $**P < 0.01$, $***P < 0.001$. Experiments are representative of duplicate repeats.

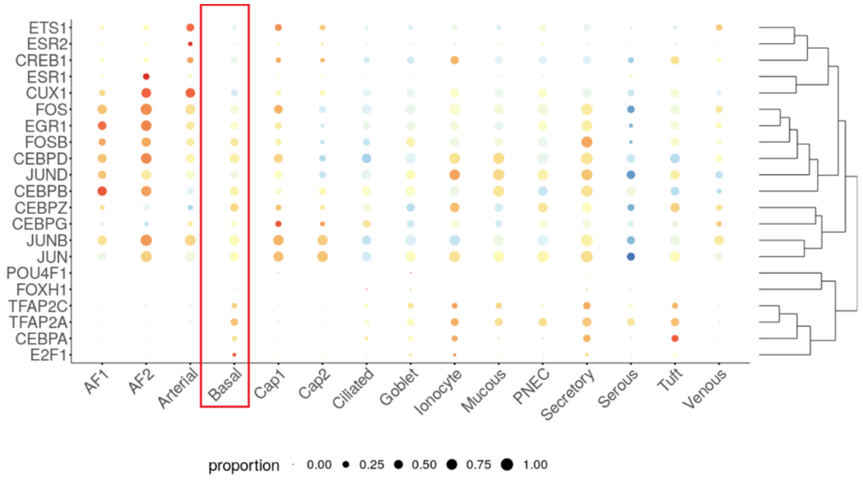
ETS1
ESR2
CREB1
ESR1
CUX1
FOS
EGR1
FOSB
CEBPD
JUND
CEBPB
CEBPZ
CEBPG
JUNB
JUN
POU4F1
FOXH1
TFAP2C
TFAP2A
CEBPA
E2F1
AF1
AF2
Arterial
Basal
Cap1
Cap2
Ciliated
Goblet
Ionocyte
Mucous
PNEC
Secretory
Serous
Tuft
Venous
proportion
0.00
0.25
0.50
0.75
1.00
expression
-2
0
2

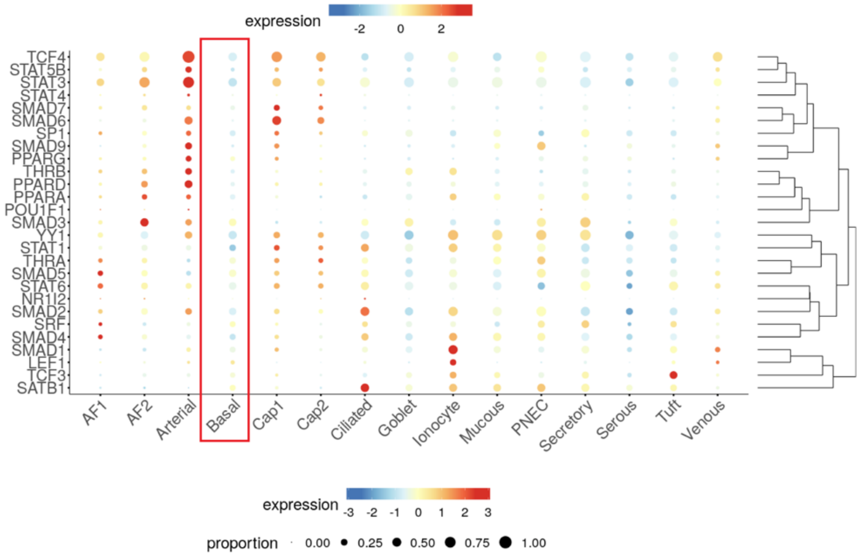
TCF4
STAT5B
STAT3
STAT4
SMAD7
SMAD6
SP1
SMAD9
PPARG
THRB
PPARD
PPARA
POU1F1
SMAD3
YY1
STAT1
THRA
SMAD5
STAT6
NR1I2
SMAD2
SRF
SMAD4
SMAD1
LEF1
TCF3
SATB1
AF1
AF2
Arterial
Basal
Cap1
Cap2
Ciliated
Goblet
Ionocyte
Mucous
PNEC
Secretory
Serous
Tuft
Venous
expression
-3
-2
-1
0
1
2
3
proportion
0.00
0.25
0.50
0.75
1.00

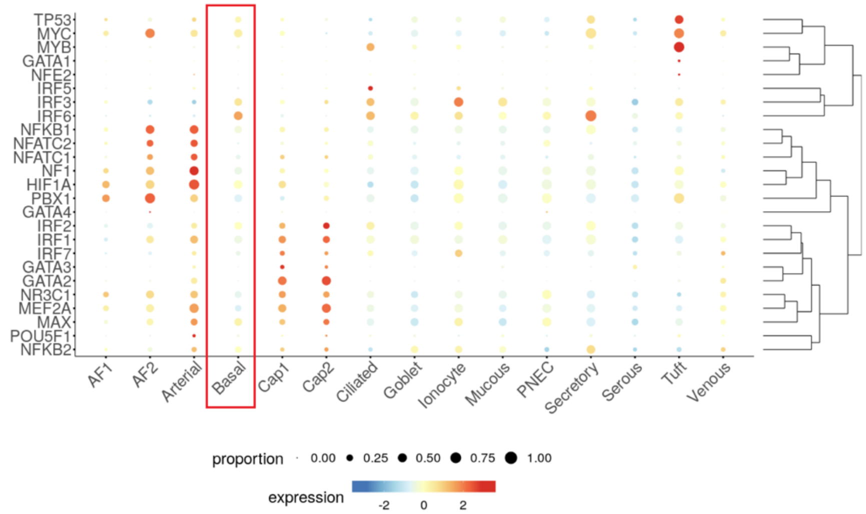
TP53
MYC
MYB
GATA1
NFE2
IRF5
IRF3
IRF6
NFKB1
NFATC2
NFATC1
NF1
HIF1A
PBX1
GATA4
IRF2
IRF1
IRF7
GATA3
GATA2
NR3C1
MEF2A
MAX
POU5F1
NFKB2
AF1
AF2
Arterial
Basal
Cap1
Cap2
Ciliated
Goblet
Ionocyte
Mucous
PNEC
Secretory
Serous
Tuft
Venous
proportion
0.00
0.25
0.50
0.75
1.00
expression
-2
0
2

**Supplementary Figure 4. LungMap single-cell TF expression for candidates from TF binding array.** Bubble plots generated for transcription factors relevant to the TF array in Figure 4A using the LungMap shinycell explorer. This is based on data generated by the LungMap Consortium [U01HL122642] and downloaded from www.lungmap.net on December 14, 2023. Bubble plots displaying TF expression levels for human structural lung cells are shown for comparison to basal cells highlighted by red boxes.

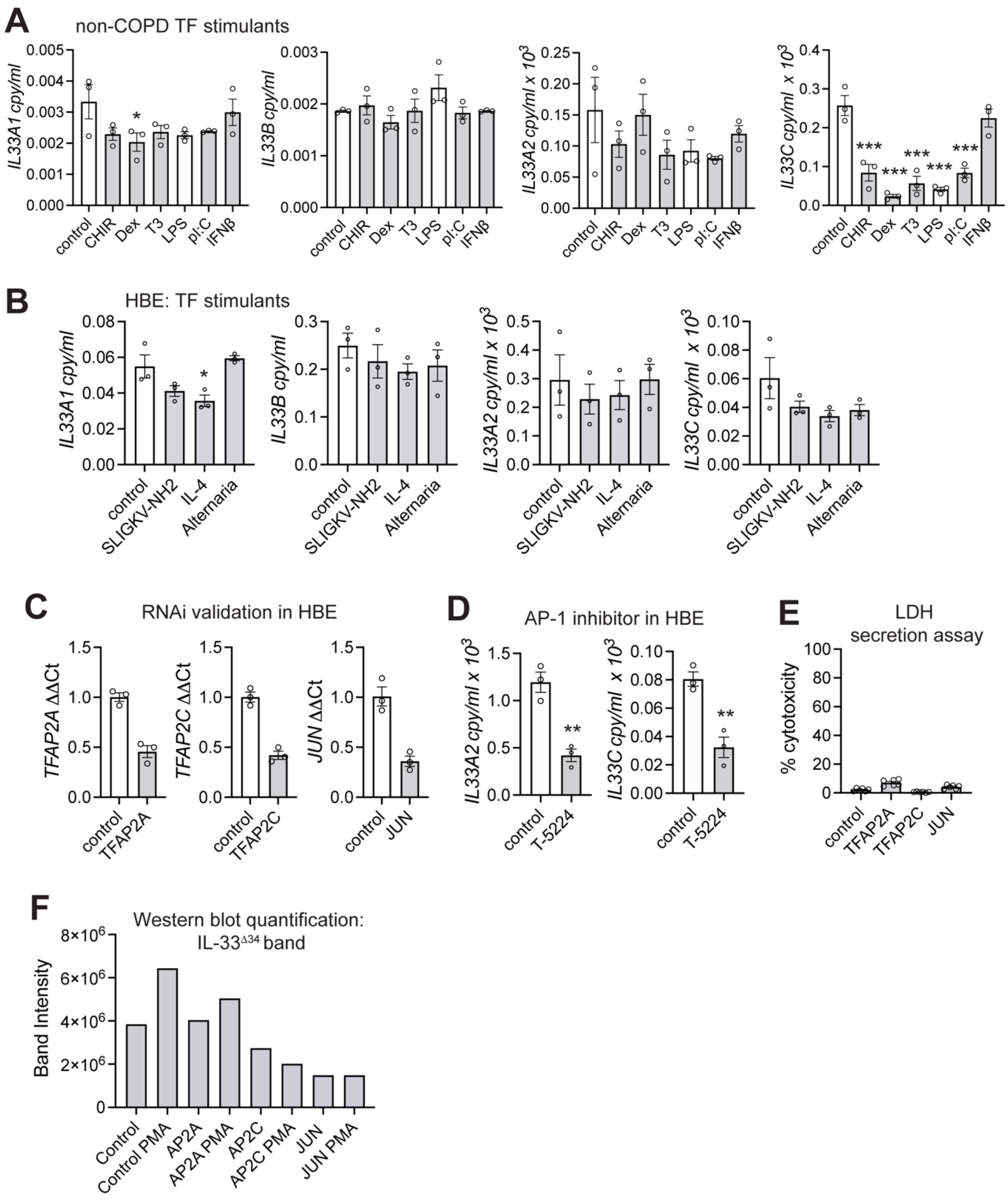


**Supplementary Figure 5. HBE and basal cell response to TF activators and inhibitors. A)** Follow up *IL33* expression analysis for TFs with > 30% inhibition in array performed in non-COPD airway basal cells, working concentrations are reported in

Supplementary Table 4. **B)** Additional TF activators tested for *IL33* expression to address endogenous disease-relevant upstream signals mimicked by PMA in HBE cells. **C)** Validation of RNAi with relative expression for AP-1 (*JUN*) and AP-2 (*TFAP2A, TFAP2C*) transcription factors under control and shRNA knockdown conditions. **D)** Expression levels for *IL33A2* and *IL33C* under conditions of AP-1 inhibitor treatment. **E)** LDH release assay to demonstrate minimal cellular toxicity in setting of IL-33 secretion assay post-PMA treatment in Figure 4E. **F)** Quantitative summary of band intensity for lower ~24 kDa band on anti-IL-33 CTD western blot under RNAi conditions in HBE cells treated with and without PMA. Statistical analysis: one-way ANOVA (A, B), *P*-value limits: **P* < 0.05, ***P* < 0.01, ****P* < 0.001. Data shown is representative of experiments performed in duplicate.

**Supplementary Table 1.** Clinical characteristics of non-COPD and severe (GOLD Stage IV) COPD lung specimens.

| Characteristics | Non-COPD | COPD |
|---|---|---|
| Number per group | 18 | 20 |
| Mean age (range) | 32.13 (18-67) | 61.4 (52-70) |
| Male:Female | 7:2:9 | 7:13 |
| FVC (L) | – | 1.97 (0.21) |
| FEV1 (L) | – | 0.52 (0.04) |
| $FEV_1$/FVC (%) | – | 24.95 |
| Pack-years (range) | 3.75 (0-15) | 42.74 (10-96) |

**Supplementary Table 2:** List of reagents and materials.

| Reagent | Supplier | Reference/Iden |
|---|---|---|
| **Antibodies** | | |
| Human IL-33 rabbit polyclonal (1:50 IF) | Sigma | HPA024426 |
| Human IL-33 (CTD) goat monoclonal (1:1000 WB) | R&D systems | MAB36253 |
| Human IL-33 (NTD) goat polyclonal (1 ug/mL) | R&D systems | AF4810 |
| Human IL-33 mouse monoclonal (Nessy-1) (1:100 IHC) | Enzo | ALX-804-840PF |
| AP2 alpha monoclonal (3B5) (1:100 IHC) | Invitrogen | MA1-872 |
| AP2 gamma monoclonal (3A11A5) (1:100 IHC) | Invitrogen | MA5-31928 |
| Phospho-AP2-gamma (Serr434) polyclonal (1:50 IHC) | Invitrogen | PA5-105357 |
| c-Jun monoclonal | Invitrogen | MA5-15881 |
| c-Fos monoclonal | Invitrogen | MA5-15055 |
| | | |
| IF = immunofluorescence, WB = western blot | | |
| | | |
| **Commercial lentiviruses** | | |
| TFAP2A shRNA | Santa Cruz | sc-105074-V |
| TFAP2C shRNA | Santa Cruz | sc-29696-V |
| JUN shRNA | Santa Cruz | sc-29223-V |
| Scrambled control shRNA | Santa Cruz | sc-108080 |

| | | |
|---|---|---|
| **Biological Samples** | | |
| Primary human basal cells | BJH | N/A |
| Control and COPD lung tissue | BJH | N/A |
| | | |
| **Chemicals, Peptides, and Recombinant Proteins** | | |
| Dexamethasone | Sigma | D4902 |
| Interferon-β | R&D Systems | 8499-IF |
| CHIR99021 | Stem Cell | 72052 |
| Lipopolysaccharide (LPS) | Millipore-Sigma | LPS25 |
| Poly I:C (lipofectamine transfected) | Enzo | ALX-746-021 |
| IL-4 | R&D systems | BT-004 |
| Cobalt chloride | Sigma | C8861 |
| SLIGKV-NH2 | Tocris | 3010 |
| *Alternaria alternata* extract | Greer | XPM1D3A2.5 |
| Triiodothyronine (T3) | Sigma | T6397 |
| T-5224 (AP-1 inhibitor) | Tocris | 7317 |
| Phorbol-12-myristate 13-acetate (PMA) | Tocris | 1201 |
| | | |
| **Commercial Assays** | | |
| TF binding array | Signosis | FA-2001 |
| | | |
| **Cell Lines** | | |

| | | |
|---|---|---|
| HBE-1 | UNC Marisco Lung Institute | Yankaskas et al, 1993 |
| Beas2B | Millipore | 95102433 |
| 16HBE14o- | Millipore | SCC150 |
| | | |
| **Oligonucleotides** | | |
| **Cloning primers** | | |
| IL33A promoter 5'Kpn | GGGGTACCGCAAAATTTCTCATGAGG | |
| IL33A promoter 3'Xho | CCGCTCGAGCTTTTTATTAGCCAAAGTTTGG | |
| IL33B promoter 5'Kpn | GGGGTACCCCTTCTATATTATATGTTATGC | |
| IL33B promoter 3'Xho | CCGCTCGAGAAGGCAGTTACATCCCAGTTC | |
| IL33A2 promoter 5' Kpn | GGGGTACCCCCCTCCCTCCTATTTATTGC | |

| | |
|---|---|
| IL33A2 promoter 3' Xho | CCGCTCGAGAGC AGAAAATAAAGG GATGAAGTAATG C |
| IL33C promoter 5'Kpn | GGGGTACCTGCT TGTCCTACTAG |
| hIL33C promoter 3'Xho | CCGCTCGAGAGA GAGGAAATCCG |
| IL33C 5'Nde | GGGAATTCGACT TACTCACTGCTG CCTTCC |
| IL33A2 5'Nde | GGGAATTCACGA GATGGAGAGAGG GTGAGTAGGAGC AAAATTTCTCATG AGAATACTGAAAA ATGTAC |
| IL33A 5'Nde | GGGAATTCCATA TGACACAGAGCT GCAGCTCTTCAG GGAAG |

| | |
|---|---|
| IL33B 5’Nde | GGGAATTCCATATGAAATACTACAATTGCTGACTACAGGAAACC |
| IL33 3’Xho | CCGCTCGAGTCATCAGTTTCAGAGA |
| | |
| **qPCR primer-probe sets** | |
| | |
| IL33 Δ34 specific | |
| Forward Primer | CACAGCAAAGTGGAAGAACAC |
| Probe | AGCTTGAAACACAAGGCTTTGCTTGC |
| Reverse Primer | TACTCTGTAATAGGTGAAATTCTTCCC |
| | |
| IL33B Δ34 specific | |
| Forward Primer | CATCATCTGAGACCAGCACTT |

| | |
|---|---|
| Probe | TGGAAGAACACA<br>GCAAGCAAAGCC |
| Reverse Primer | TAGGTGAAATTCT<br>TCCCAGCTT |
| | |
| IL33A1 Δ34 specific | |
| Forward Primer | AGCTCTTCAGGG<br>AAGAAATCAAA |
| Probe | TGGAAGAACACA<br>GCAAGCAAAGCC |
| Reverse Primer | AGGTGAAATTCTT<br>CCCAGCTT |
| | |
| IL33 full specific | |
| Forward Primer | GGTGGTTTCTCT<br>CCTAAAGTAACA<br>G |
| Probe | TTTATGAAGCTCC<br>GCTCTGGCCTT |
| Reverse Primer | CCCAACAGAAGG<br>CCAAAGAA |
| | |
| IL33A | |

| | |
|---|---|
| Forward Primer | CAGCTCTTCAGG GAAGAAATCA |
| Probe | TGCTTGCTGTGTT CTTCCACTTTGC |
| Reverse Primer | TCCCAGCTTGAA ACACAAGG |
| | |
| IL33A2 | |
| Forward Primer | CAGATGCCAAAC GAGATGGA |
| Probe | AGTTTGGCAGAG ACCCAAATTGCC |
| Reverse Primer | TGTGTTCGGAGG AGTCTGTA |
| | |
| IL33B | |
| Forward Primer | AATACTACAATTG CTGACTACAGGA |
| Probe | ACCTCATCA/ZEN/ TCTGAGACCAGC ACT |
| Reverse Primer | CTTGCTGTGTTCT TCCACTTTG |

| | |
|---|---|
| IL33C | |
| Forward Primer | CTCACCAGATGC CAGCATAATA |
| Probe | CCAACAGAAGGC CAAAGAAGTTTG CC |
| Reverse Primer | GAGCGGAGCTTC ATAAAGTACA |
| | |
| TFAP2A IDT predesigned assay | Hs.PT.58.5602 |
| | |
| TFAP2C IDT predesigned assay | Hs.PT.58.2610271 0 |
| | |
| JUN IDT predesigned assay | Hs.PT.58.2509471 4.g |
| FOS IDT predesigned assay | |
| | |
| **SYBR Green IDT PrimeTime qPCR Primers** | |
| IL-33 A1 Set | |

| | |
|---|---|
| Primer 1 | 5'-CGTCCTCCCTCCATCTGAATA-3' |
| Primer 2 | 5'-TGGACAGTGCTGAAGTTGAAG-3' |
| | |
| IL-33 A2 Set | |
| Primer 1 | 5'-TCATCTGCTACAAGCCATAGTG-3' |
| Primer 2 | 5'-CGCTTGCTTCACTGCTTTATC-3' |
| | |
| IL-33 B Set | |
| Primer 1 | 5'-TCCCAAAGTGCTGGGATTAC-3' |
| Primer 2 | 5'-CACTGCCTAGAGCCTATATTGC-3' |
| | |

| | |
|---|---|
| IL-33 C Set | |
| Primer 1 | 5'-AACCAAACCACTCCCATGATAA-3' |
| Primer 2 | 5'-CTATCTCAGCTCACTGCAACC-3' |
| | |
| IL-33 Intron 1 enhancer set | |
| Primer 1 | 5'-CACAGTGCAGAGGAGGAAATAC-3' |
| Primer 2 | 5'-GTCCTGTGGCTGCATACAA-3' |
| | |
| IL-33 A1 upstream enhancer set | |
| Primer 1 | 5'-AGATGGAGTCTTGCTTCATCAC-3' |

| | |
|---|---|
| Primer 2 | 5'-<br>GAGGCAGGAGAA<br>TTGCTTGA-3' |

**Supplementary Table 3:** List of reagents and working concentrations for *IL33* expression screening.

| Agent | Working Concentration | Source |
|---|---|---|
| PMA | 20ng/ml | Tocris |
| ionomycin | 500 ng/ml | Tocris |
| ATP | 10 uM | Sigma |
| AMP | 10 uM | Sigma |
| epinephrine | 27 uM | Sigma |
| uric acid | 6 uM | Sigma |
| DAPT | 1 uM | Sigma |
| DLK1 | 1000 ng/ml | R&D systems |
| HGF | 100 ng/ml | Peprotech |
| R-Spondin | 5 ng/ml | Stem Cell technologies |
| retinoic acid | 50 nM | Sigma |
| IFN-beta | 100 ng/ml | R&D systems |
| IFN-gamma | 100 ng/mL | R&D systems |
| IL-13 | 100 ng/ml | R&D systems |
| IL-17A | 100 ng/ml | R&D systems |
| IL-1a | 100 ng/ml | R&D systems |
| IL-33 | 100 ng/ml | R&D systems |
| IL-4 | 100 ng/ml | R&D systems |

| | | |
|---|---|---|
| TNF | 100 ng/ml | Sigma |
| trefoil factor 2 | 100 ng/ml | Peprotech |
| trefoil factor 1 | 100 ng/ml | Peprotech |
| pI:C- lipofectamine transfected | 1 ug/ml | Sigma |
| LPS | 1 ug/ml | Sigma |
| Alternaria alternata extract | 20 microgram/ml | Greer |
| House dust mite extract | 20 microgram/ml | Greer |
| PBS | | Sigma |

**Supplementary Table 4:** List of reagents and working concentrations for follow up on hits from TF array.

| Agent | Working Concentration | Source |
|---|---|---|
| PMA | 20ng/ml | Tocris |
| T5524 (AP-1 inhibitor) | 10 μM | Tocris |
| dexamethasone | 100 nM | Tocris |
| Interferon-β | 10 μM | R&D systems |
| Poly I:C (transfected) | 1 μg/ml | Enzo |
| LPS | 1 μg/ml | Sigma |
| Cobalt chloride | 50 μM | Sigma |
| IL-4 | 100 ng/ml | R&D systems |
| CHIR99021 | 1000 ng/ml | Stem Cell Technologies |
| T3 | 100 nM | Sigma |
| Alternaria extract | 20 μg/ml | Greer |
| SLIGKV-NH2 | 100 μM | Tocris |
