# Supplementary material for: Activator protein transcription factors coordinate human IL-33 expression from noncanonical promoters in chronic airway disease
Source: JCI Insight. 2024 Mar 8;9(5):e174786. doi: 10.1172/jci.insight.174786 (PMC10972587; doi:10.1172/jci.insight.174786)
Supplement: Unedited blot and gel images [file jciinsight-9-174786-s207.pptx]

## Slide 1
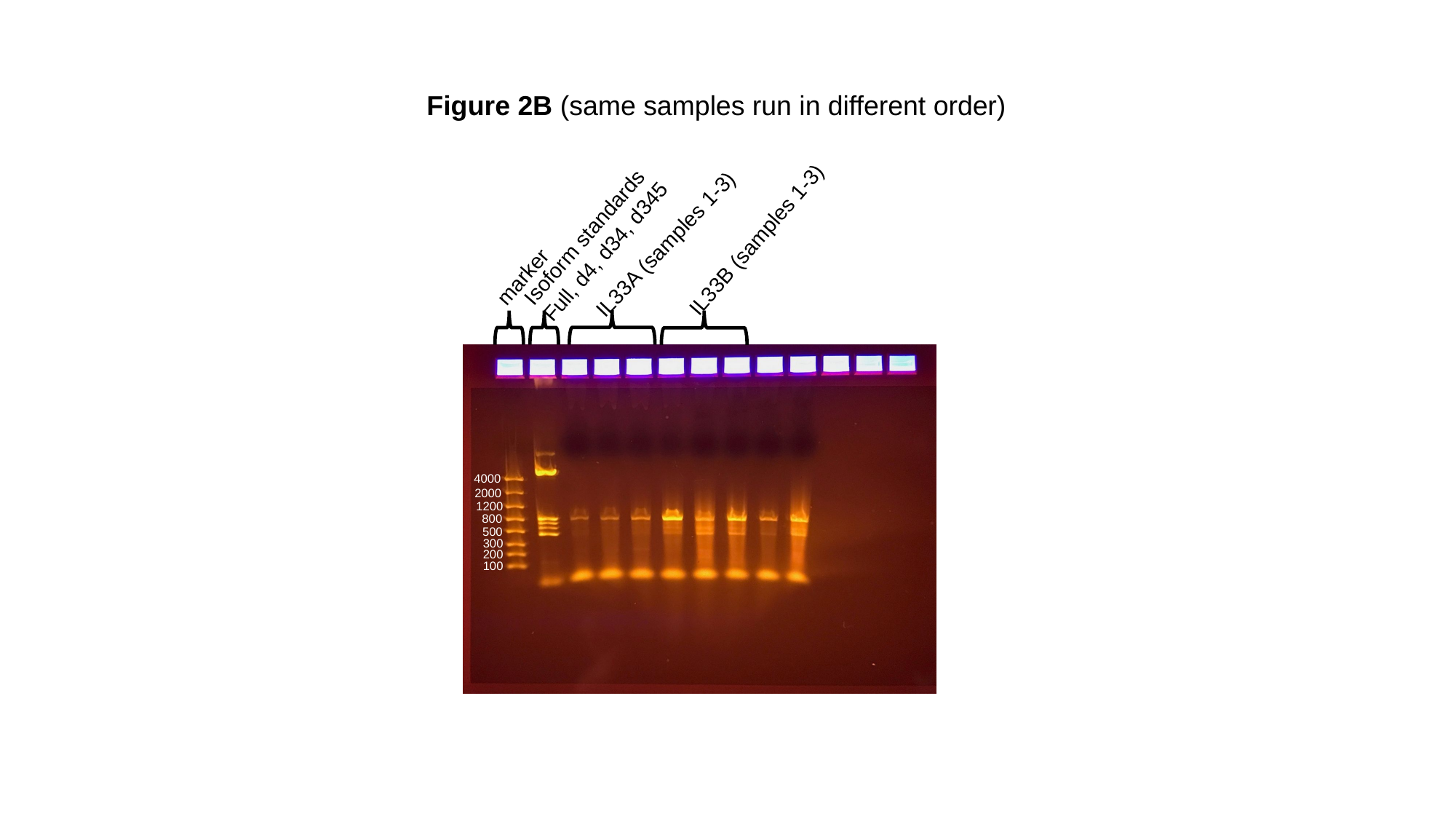

Figure 2B (same samples run in different order)
Isoform standards
Full, d4, d34, d345
IL33B (samples 1-3)
IL33A (samples 1-3)
marker
4000
2000
1200
800
500
300
200
100

## Slide 2
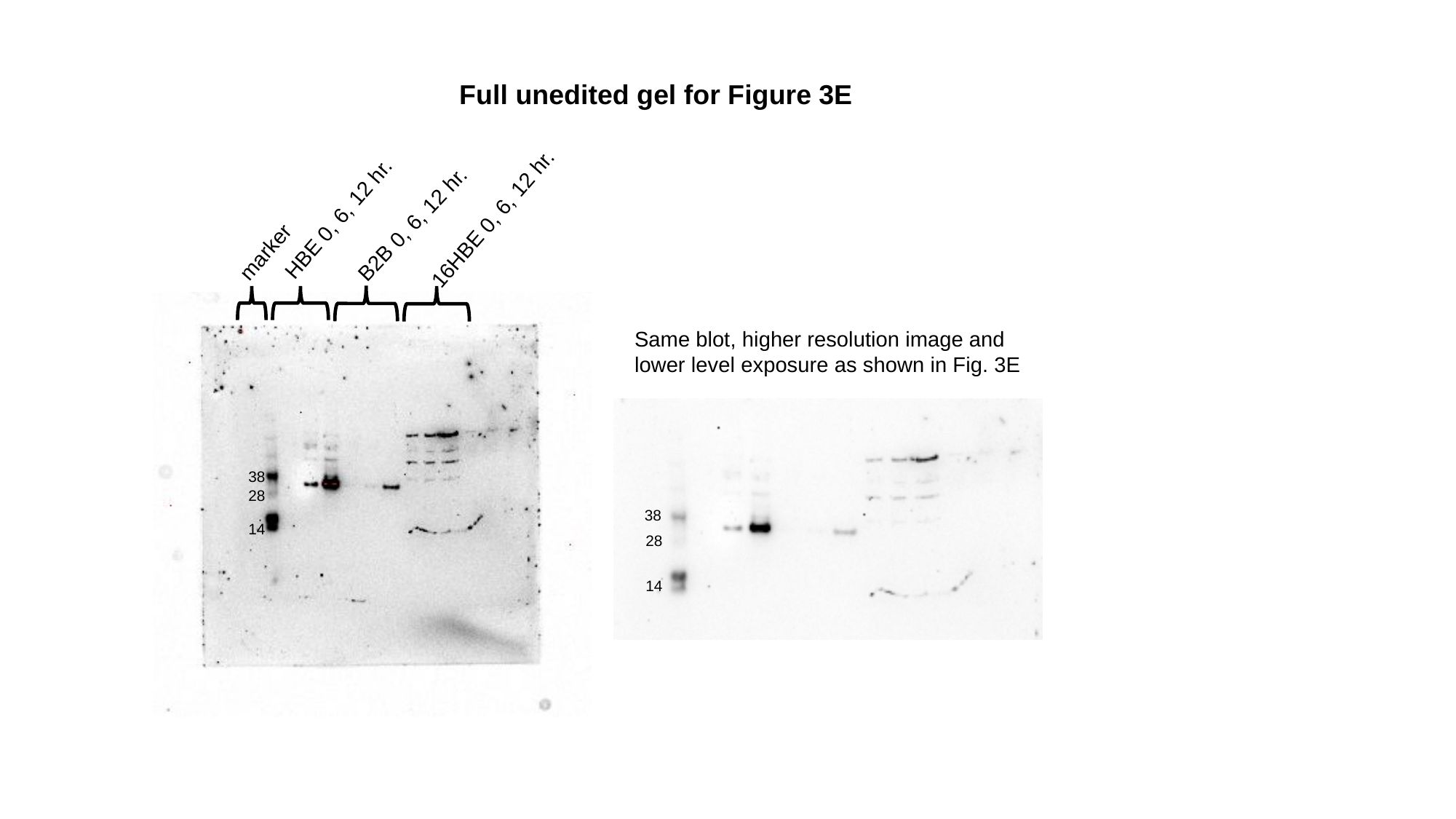

Full unedited gel for Figure 3E
16HBE 0, 6, 12 hr.
HBE 0, 6, 12 hr.
B2B 0, 6, 12 hr.
marker
Same blot, higher resolution image and
lower level exposure as shown in Fig. 3E
38
28
38
14
28
14

## Slide 3
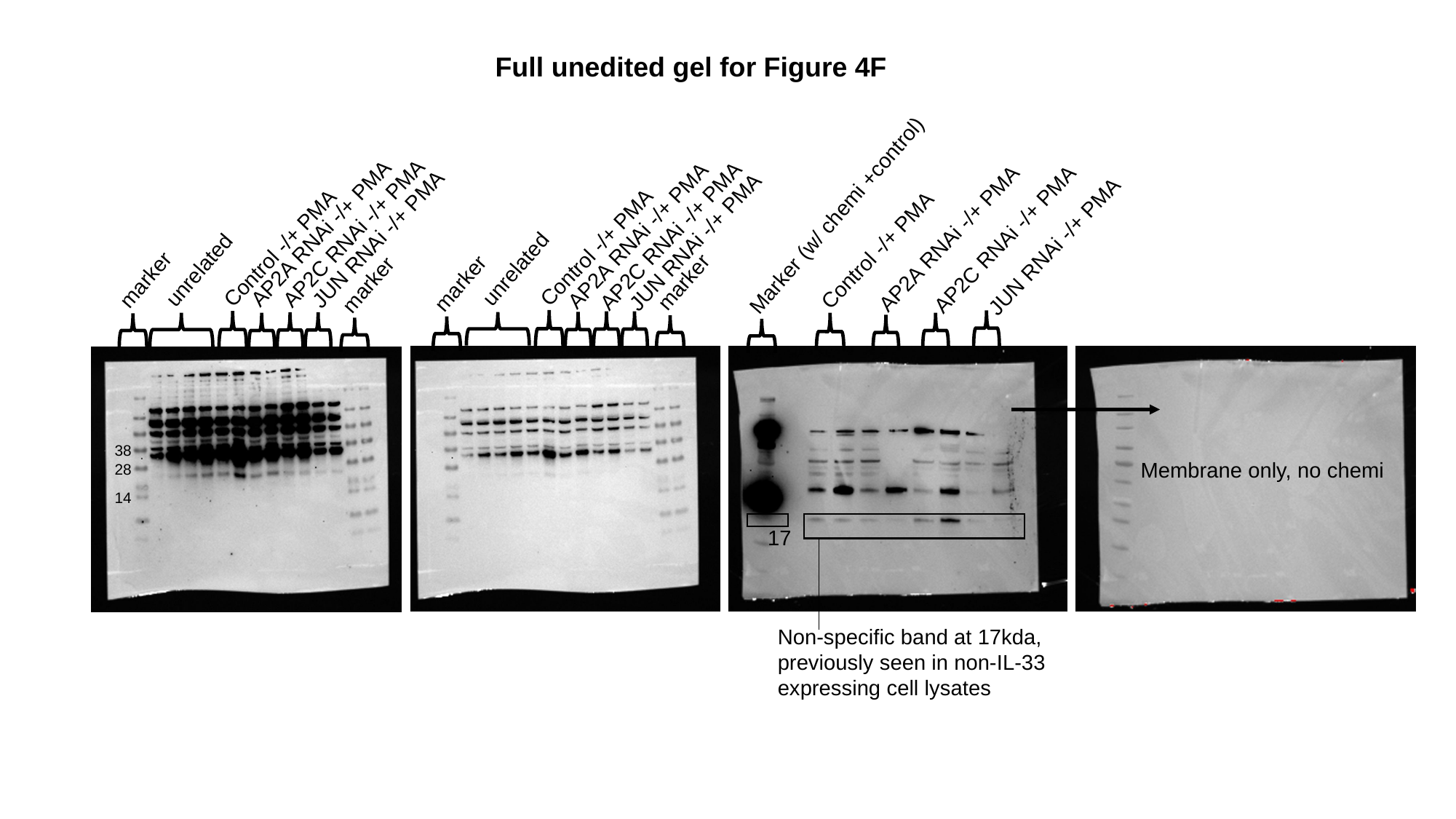

Full unedited gel for Figure 4F
Marker (w/ chemi +control)
AP2A RNAi -/+ PMA
AP2C RNAi -/+ PMA
JUN RNAi -/+ PMA
AP2A RNAi -/+ PMA
AP2C RNAi -/+ PMA
JUN RNAi -/+ PMA
AP2A RNAi -/+ PMA
AP2C RNAi -/+ PMA
JUN RNAi -/+ PMA
Control -/+ PMA
Control -/+ PMA
Control -/+ PMA
unrelated
unrelated
marker
marker
marker
marker
38
Membrane only, no chemi
28
14
17
Non-specific band at 17kda, previously seen in non-IL-33 expressing cell lysates
